# Distributions and trends of the global burden of COPD attributable to risk factors by SDI, age, and sex from 1990 to 2019: a systematic analysis of GBD 2019 data

**DOI:** 10.1186/s12931-022-02011-y

**Published:** 2022-04-11

**Authors:** Jiahua Zou, Tao Sun, Xiaohui Song, Ye-Mao Liu, Fang Lei, Ming-Ming Chen, Ze Chen, Peng Zhang, Yan-Xiao Ji, Xiao-Jing Zhang, Zhi-Gang She, Jingjing Cai, Yunman Luo, Ping Wang, Hongliang Li

**Affiliations:** 1 Department of Cardiology, Renmin Hospital of Wuhan University, Wuhan, 430060 China; 2 Huanggang Institute of Translational Medicine, Huanggang, China; 3 Department of Oncology, Huanggang Central Hospital, NO. 16 Kaopeng Street, Huangzhou District, Huanggang, 438021 China; 4 Institute of Model Animal, Wuhan University, Wuhan, China; 5 School of Basic Medical Science, Wuhan University, Wuhan, China; 6 Department of Cardiology, Zhongnan Hospital of Wuhan University, Wuhan, China; 7 Department of Cardiology, The Third Xiangya Hospital, Central South University, Changsha, China; 8 Medical Science Research Center, Zhongnan Hospital of Wuhan University, Wuhan, China

**Keywords:** Chronic obstructive pulmonary disease, Global disease burden, Prevention, Risk factors

## Abstract

**Background:**

Global distributions and trends of the risk-attributable burdens of chronic obstructive pulmonary disease (COPD) have rarely been systematically explored. To guide the formulation of targeted and accurate strategies for the management of COPD, we analyzed COPD burdens attributable to known risk factors.

**Methods:**

Using detailed COPD data from the Global Burden of Disease study 2019, we analyzed disability-adjusted life years (DALYs), years lived with disability (YLDs), years of life lost (YLLs), and deaths attributable to each risk factor from 1990 to 2019. Additionally, we calculated estimated annual percentage changes (EAPCs) during the study period. The population attributable fraction (PAF) and summary exposure value (SEV) of each risk factor are also presented.

**Results:**

From 1990 to 2019, the age-standardized DALY and death rates of COPD attributable to smoking and household air pollution, occupational particles, secondhand smoke, and low temperature presented consistently declining trends in almost all socio-demographic index (SDI) regions. However, the decline in YLD was not as dramatic as that of the death rate. In contrast, the COPD burden attributable to ambient particulate matter, ozone, and high temperature exposure showed undesirable increasing trends in the low- and low-middle-SDI regions. In addition, the age-standardized DALY and death rates attributable to each risk factor except household air pollution and low temperature were the highest in the low-middle-SDI region. In 2019, the COPD burden attributable to smoking ambient particulate matter, ozone, occupational particles, low and high temperature was obviously greater in males than in females. Meanwhile, the most important risk factors for female varied across regions (low- and low-middle-SDI regions: household air pollution; middle-SDI region: ambient particles; high-middle- and high-SDI region: smoking).

**Conclusions:**

Increasing trends of COPD burden attributable to ambient particulate matter, ozone, and high temperature exposure in the low-middle- and low-SDI regions call for an urgent need to implement specific and effective measures. Moreover, considering the gender differences in COPD burdens attributable to some risk factors such as ambient particulate matter and ozone with similar SEV, further research on biological differences between sexes in COPD and relevant policy-making of disease prevention are required.

**Supplementary Information:**

The online version contains supplementary material available at 10.1186/s12931-022-02011-y.

## Background

Chronic obstructive pulmonary disease (COPD), the most pervasive chronic respiratory disease and the leading contributor to the global disease burden, increased in rank from 11 to 6th in disability-adjusted life years (DALYs) among all causes between 1990 and 2019 [1, 2]. In 2019, COPD caused 3.28 million deaths and 74.43 million DALYs, 78.77 and 75.78% of which were attributable to exposure risk factors, respectively. Additionally, the prevalence and disease burden of COPD varied by sex and socioeconomic status [2]. A previous study found that low-sociodemographic index (SDI)-regions had the greatest burdens of COPD, and risk factors such as smoking and environmental pollution had the highest contributions to COPD-related mortality and DALYs [3]. However, key questions about the COPD disease burden by area, sex, and age group at the risk factor level remain unanswered, hindering the prevention and control of COPD.

According to Global Burden of Disease (GBD) study data from 1990 to 2019, the prevalence rates of risk factors changed substantially. The summary exposure values (SEVs) of smoking and household air pollution from solid fuels decreased in all SDI quintiles. In contrast, the SEV of ambient particulate matter pollution had one of the largest increases. Moreover, the SEVs of secondhand smoke and occupational particles differed greatly among different regions [4]. The changes in these top risk factors contributing to the COPD disease burden were mostly influenced by economic development and demographic shifts. Therefore, in addition to depicting the overall pattern of the COPD burden, timely research to comprehensively study the COPD burden attributable to various risk factors is needed.

In the present study, we analyzed the burdens of COPD attributable to eight risk factors by SDI, age, and sex between 1990 and 2019 to reveal the different trends and distribution features of the burden attributable to each risk factor. By comparing the levels of exposure to risk factors and disease burden attributable to each risk factor vertically and horizontally, this study provides further evidence for implementing relevant policies and strategies to prevent and control future increases in the COPD burden.

## Methods

### Data source

This study on the burden of COPD and its risk factors did not involve human subjects, and the data were retrieved from the Global Health Data Exchange GBD Results Tool (http://ghdx.healthdata.org/gbd-results-tool). The GBD study establishes a freely accessible database containing data on estimated attributable burdens obtained with standardized methods for a wide range of risk factors for all countries [4]. The GBD 2019 estimated the age- and sex-specific global burdens of 369 diseases and injuries and 87 risk factors in 204 countries and territories from 1990 to 2019 by quantifying the health loss from premature mortality and nonfatal disability [1, 4]. This study complies with Guidelines for Accurate and Transparent Health Estimates Reporting (GATHER) guidelines for reporting health estimates [5].

### Risk factors

Smoking and exposure to secondhand smoke, household air pollution from solid fuels, ambient particulate matter, ozone, occupational particles, low temperatures, and high temperatures were identified as risk factors for COPD. These risk factors from the GBD were selected based on sufficient evidence of causality, availability of exposure data, potential for behavioral and other modifications, and health policy interest [2, 4, 6]. In GBD 2019, risk-outcome pairs that have been assessed as meet the World Cancer Research Fund grades of convincing or probable evidence are included, for collating relative risk data [4, 7]. And then determining relative risks for the risk-outcome pairs. The exposure data of risk factors need to be available. For smoking, second-hand smoke, and household air pollution, the exposure data of which still mainly comes from population surveys. Ambient particulate matter pollution exposure comes from multiple sources, including satellite observations of aerosols in the atmosphere, ground measurements, chemical transport model simulations, population estimates, and land-use data. Exposure data of ozone comes from ground measurement data combined with chemical transport model estimates using Bayesian maximum entropy. Exposure data of occupational is obtained from the International Labour Organization (ILO). Exposure data of low and high temperatures (exposure to the ambient temperature that is either warmer or colder than the temperature associated with the minimum mortality risk) comes from the ERA5 reanalysis dataset from the European Centre for Medium-Range Weather Forecasts (ECMWF) [4]. The population attributable fraction (PAF) reflects the extent of the impact, which represents the proportion of an outcome in the population that would be eliminated if a risk factor were removed. In the GBD 2019, PAFs took the risk function and the distribution of exposure across individuals in each age-sex-location-year into consideration and were calculated independently by risk factor by using risk exposures, estimates of relative risk based on meta-analyses, and theoretical minimum risk levels determined for each risk-outcome pair [4, 8, 9]. In addition, exposure distributions for each risk factor were summarized using the summary exposure value (SEV), which compares the distribution of excess risk times exposure level to a population where everyone is at maximum risk.

### Statistical analyses

We calculated the estimated annual percentage changes (EAPCs) in age-standardized rates (ASRs); the EAPC is a summary and widely used measure of an ASR trend over a specific time interval and can be used to quantify the trend of the COPD burden attributable to each risk factor [10, 11]. The ASR (per 100,000 population) was calculated based on the following formula:

$$ASR = \frac{{\sum\nolimits_{i = 1}^{A} {a_{i} w_{i} } }}{{\sum\nolimits_{i = 1}^{A} {w_{i} } }} \times 100,000$$

where *a*_*i*_ denotes the *i*th age class and the number of persons (or weight) (*w*_*i*_) in the same age subgroup i of the chosen reference standard population. The natural logarithm of the ASR is assumed to be linear over time; that is, Y = α + βX + ε, where Y refers to ln (ASR), X is the calendar year, and ε is the error term. Based on this formula, β represents the positive or negative ASR trend. The EAPC was calculated as EAPC = 100 × (exp(β)-1), and its 95% confidence interval (CI) was also obtained from the linear regression model. The ASR was deemed to have increased if the EAPC estimation and the lower boundary of its 95% CI were both positive. In contrast, the ASR was deemed to have decreased if the EAPC estimation and the upper boundary of its 95% CI were both negative.

In addition, we analyzed risk-attributable DALY, death, years lived with disability (YLD), and years of life lost (YLL) rates per 100,000 population with 95% uncertainty intervals (UIs) based on the 2.5th and 97.5th values of the ordered 1000 draws in both sexes and in each sex in the different SDI regions between 1990 and 2019. The SDI, an indicator of a country’s lag-distributed income per capita, average years of schooling, and fertility rate in females under the age of 25 years, comprehensively reflects the degree of development. Countries and territories were categorized into five quintiles by SDI (low, low-middle-, middle-, high-middle, and high-SDI regions) [12]. (Additional file 1: Table S5) We also analyzed these measures in both sexes in different age groups (17 age groups, from < 20 to > 95 years, with 5-year intervals). For all analyses in our study, except the age subgroups analysis, all rates were age-standardized using the GBD standard and are reported per 100,000 population.

Spearman’s rank-order correlation was used to measure the intensity and direction of the correlations between the PAFs of age-standardized DALY caused by each risk factor and the SDI, and a two-tailed p-value was calculated; a p-value less than 0.05 was regarded as significant. All statistical analyses were performed using the R program (Version 4.0.4, R core team).

## Results

### Overall impact of risk factors on COPD burden

**Fig. 1:**
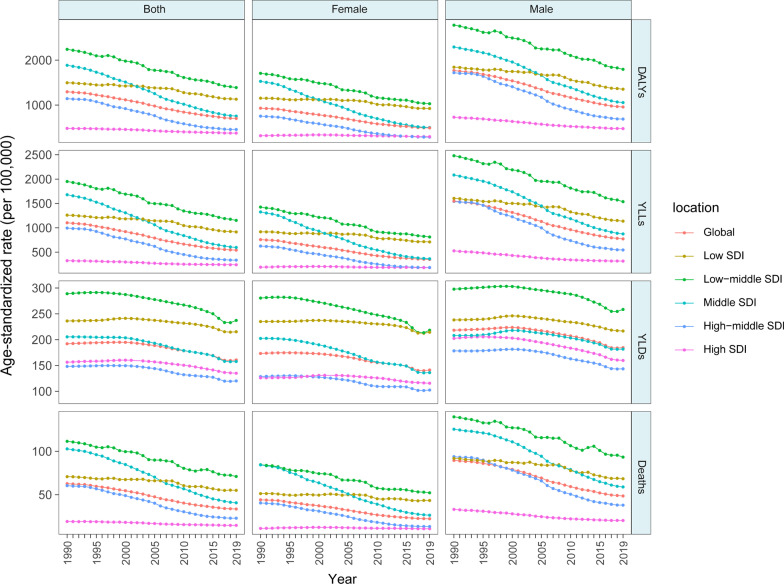
Age-standardized rates of DALY, YLL, YLD and death attributable to all risk factors of chronic obstructive pulmonary disease, 1990 to 2019. *DALY* disability-adjusted life year, *YLL* year of life lost, *YLD* year lived with disability

From 1990–2019, the global age-standardized DALY, YLL, YLD and death rates of COPD attributable to risk factors showed steady downward trends (Fig. 1). However, YLD declined at a slower rate than YLL over time and even increased in the recent years, suggesting that improvements in standards of living and quality of life need to be strengthened for COPD patients (Fig. 1, Additional file 1: Tables S2, S3). These trends were similar in the male and female populations, but as shown in Fig. 1, the overall disease burden of COPD was lower in females than in males. The global trend of the age-standardized DALY rate declined relatively slower from 2010 to 2019 (EAPC = − 2.12[− 2.30–− 1.93]) than from 2000 to 2009 (EAPC = − 2.85[− 2.98–− 2.71]) (Fig. 1, Table 1).

**Table 1:** The temporal trends of age-standardized DALY rate attributed to risk factors across different SDI regions, 1990 to 2019

		All risk factors	Smoking	Household air pollution	Occupational particles	Ambient particulate matter	Ambient ozone pollution	Secondhand smoke	High temperature	Low temperature
Global EAPC(95%CI)	1990–1999	− 1.46 (− 1.67 – − 1.25)	− 0.87 (− 1.13 – − 0.61)	− 4.03 (− 4.34–− 3.72)	− 1.41 (− 1.63 – − 1.19)	0.47 (0.37–0.56)	0.46 (0.23–0.70)	− 1.86 (− 2.20–− 1.51)	15.04 (8.20–22.30)	− 2.52 (− 3.33–− 1.71)
	2000–2009	− 2.85 (− 2.98–− 2.71)	− 2.94 (− 3.11–− 2.77)	− 5.57 (− 5.82–− 5.32)	− 2.79 (− 2.94–− 2.63)	− 1.09 (− 1.29–− 0.88)	− 0.60 (− 1.31–0.11)	− 3.41 (− 3.57–− 3.25)	2.39 (− 0.31–5.16)	− 4.25 (− 4.69–− 3.81)
	2010–2019	− 2.12 (− 2.30–− 1.93)	− 2.24 (− 2.42–− 2.06)	− 6.19 (− 6.42–− 5.96)	− 2.04 (− 2.26–− 1.81)	− 0.91 (− 1.46–− 0.34)	− 1.26 (− 2.11–− 0.40)	− 1.83 (− 2.14–− 1.52)	2.06 (− 0.10–4.26)	− 2.99 (− 3.53–− 2.44)
Low SDI EAPC (95%CI)	1990–1999	− 0.45 (− 0.61–− 0.30)	− 0.45 (− 0.62–− 0.28)	− 1.06 (− 1.21–− 0.92)	− 0.44 (− 0.60–− 0.28)	1.10 (0.37–1.83)	1.07 (0.30–1.85)	− 0.59 (− 0.74–− 0.43)	–	− 0.55 (− 1.15–0.06)
	2000–2009	− 0.77 (− 0.98–− 0.56)	− 0.72 (− 0.93–− 0.51)	− 2.13 (− 2.43–− 1.83)	− 0.52 (− 0.71–− 0.33)	2.35 (2.03–2.68)	1.73 (0.56–2.90)	− 1.14 (− 1.35–− 0.94)	20.89 (10.16–32.66)	− 1.18 (− 1.83–− 0.52)
	2010–2019	− 1.47 (− 1.65–− 1.29)	− 1.59 (− 1.73–− 1.45)	− 4.09 (− 4.42–− 3.77)	− 1.11 (− 1.29–− 0.94)	1.94 (0.70–3.20)	1.51 (1.08–1.94)	− 0.35 (− 0.5–− 0.21)	3.65 (− 0.23–7.68)	− 1.01 (− 2.19–0.19)
Low− middle SDI EAPC (95%CI)	1990–1999	− 1.11 (− 1.33–− 0.89)	− 0.89 (− 1.14–− 0.64)	− 2.54 (− 2.81–− 2.27)	− 0.98 (− 1.22–− 0.73)	1.23 (0.58–1.89)	1.49 (0.90–2.08)	− 1.14 (− 1.33–− 0.94)	25.14 (9.75–42.69)	− 2.01 (− 2.72–− 1.30)
	2000–2009	− 1.91 (− 2.25–− 1.57)	− 2.00 (− 2.33–− 1.68)	− 4.46 (− 4.81–− 4.10)	− 1.58 (− 1.92–− 1.24)	1.49 (0.92–2.06)	0.55 (− 0.52–1.63)	− 2.11 (− 2.39–− 1.83)	3.07 (0.43–5.78)	− 2.68 (− 3.19–− 2.17)
	2010–2019	− 1.71 (− 1.89–− 1.53)	− 1.85 (− 2.03–− 1.67)	− 6.08 (− 6.18–− 5.98)	− 1.65 (− 1.79–− 1.51)	1.57 (0.36–2.79)	1.35 (1.04–1.65)	− 1.18 (− 1.35–− 1.01)	2.67 (0.34–5.05)	− 2.77 (− 3.55–− 1.98)
Middle SDI EAPC (95%CI)	1990–1999	− 2.20 (− 2.48–− 1.92)	− 1.05 (− 1.33–− 0.75)	− 6.20 (− 6.78–− 5.62)	− 2.05 (− 2.33–− 1.76)	0.79 (0.66–0.92)	− 0.11 (− 0.50–0.29)	− 2.57 (− 3.05–− 2.08)	7.33 (− 1.99–17.53)	− 3.14 (− 3.98–− 2.29)
	2000–2009	− 4.06 (− 4.30–− 3.83)	− 3.95 (− 4.25–− 3.65)	− 9.01 (− 9.68–− 8.34)	− 4.01 (− 4.29–− 3.74)	− 2.04 (− 2.34–− 1.73)	− 1.09 (− 1.90–− 0.28)	− 4.48 (− 4.71–− 4.25)	− 1.04 (− 5.36–3.47)	− 5.55 (− 6.07–− 5.02)
	2010–2019	− 3.41 (− 3.73–− 3.08)	− 3.45 (− 3.76–− 3.14)	− 10.03 (− 10.17–− 9.90)	− 3.28 (− 3.63–− 2.93)	− 2.77 (− 3.13–− 2.41)	− 5.11 (− 7.04–− 3.15)	− 3.02 (− 3.43–− 2.60)	− 0.54 (− 4.38–3.45)	− 4.34 (− 5.07–− 3.61)
High-middle SDI EAPC (95%CI)	1990–1999	− 2.23 (− 2.76–− 1.70)	− 1.40 (− 2.02–− 0.77)	− 7.83 (− 8.53–− 7.12)	− 2.26 (− 2.77–− 1.76)	− 0.69 (− 1.08–− 0.30)	− 1.05 (− 1.79–− 0.31)	− 2.52 (− 3.02–− 2.02)	− 0.72 (− 7.97–7.11)	− 3.18 (− 4.21–− 2.14)
	2000–2009	− 4.62 (− 5.08–− 4.16)	− 4.65 (− 5.14–− 4.16)	− 12.36 (− 13.34–− 11.36)	− 4.76 (− 5.27–− 4.25)	− 3.10 (− 3.59–− 2.61)	− 2.88 (− 3.63–− 2.12)	− 4.64 (− 5.11–− 4.17)	− 6.21 (− 9.49–− 2.81)	− 5.57 (− 6.20–− 4.93)
	2010–2019	− 2.76 (− 3.23–− 2.28)	− 2.71 (− 3.19–− 2.24)	− 10.33 (− 10.90–− 9.76)	− 2.80 (− 3.32–− 2.27)	− 3.21 (− 3.59–− 2.83)	− 3.07 (− 4.60–− 1.51)	− 2.71 (− 3.34–− 2.09)	− 0.05 (− 6.22–6.52)	− 3.62 (− 4.37–− 2.85)
High SDI EAPC (95%CI)	1990–1999	− 0.46 (− 0.57–− 0.35)	− 0.55 (− 0.67–− 0.44)	− 10.75 (− 11.26–− 10.24)	− 0.50 (− 0.62–− 0.39)	− 1.41 (− 1.53–− 1.28)	1.57 (0.89–2.26)	− 1.33 (− 1.48–− 1.19)	5.72 (1.23–10.40)	− 0.85 (− 1.54–− 0.15)
	2000–2009	− 1.32 (− 1.42–− 1.22)	− 1.38 (− 1.47–− 1.29)	− 11.96 (− 12.17–− 11.76)	− 1.16 (− 1.25–− 1.06)	− 2.79 (− 2.90–− 2.68)	− 1.90 (− 2.73–− 1.06)	− 2.68 (− 2.82–− 2.54)	1.49 (− 1.77–4.86)	− 1.60 (− 2.06–− 1.13)
	2010–2019	− 0.84 (− 0.93–− 0.75)	− 0.96 (− 1.04–− 0.89)	− 7.13 (− 7.65–− 6.60)	− 0.44 (− 0.51–− 0.37)	− 2.65 (− 2.99–− 2.31)	− 1.96 (− 2.60–− 1.32)	− 0.61 (− 0.77–− 0.45)	− 0.52 (− 4.51–3.64)	− 0.85 (− 1.27–− 0.44)

*DALY* disability-adjusted life year, *SDI* socio-demographic index, *EAPC* estimated annual percentage change, *CI* confidence interval

In 2019, among the five SDI quintiles, the low-middle-SDI region had the largest age-standardized DALY rate of COPD attributable to risk factors (1389.36 [1188.59–1566.23]), followed by the low-SDI region (1130.36 [966.49–1283.22]), and the high-SDI region had the lowest disease burden (376.21 [344.45–405.81]) (Additional file 1: Table S1). In the last decade, the largest decreased in the age-standardized DALY rate occurred in countries in the middle-SDI quintile (EAPC = − 3.41 [− 3.73–− 3.08]), and the smallest change occurred in countries in the high-SDI quintile (EAPC = − 0.84 [− 0.93–− 0.75]) (Fig. 1, Table 1). Additionally, the age-standardized YLL rate of COPD due to all risk factors also significantly decreased (1990–2009: EAPC = − 1.76 [− 2.02–− 1.51]); 2000–2009: EAPC = − 3.31 [− 3.47–− 3.15); 2010–2019: EAPC = − 2.32 [− 2.55–− 2.10]) (Fig. 1, Additional file 1: Table S2).

### Contributions of risk factors to the COPD burden by SDI, sex and age

**Fig. 2:**
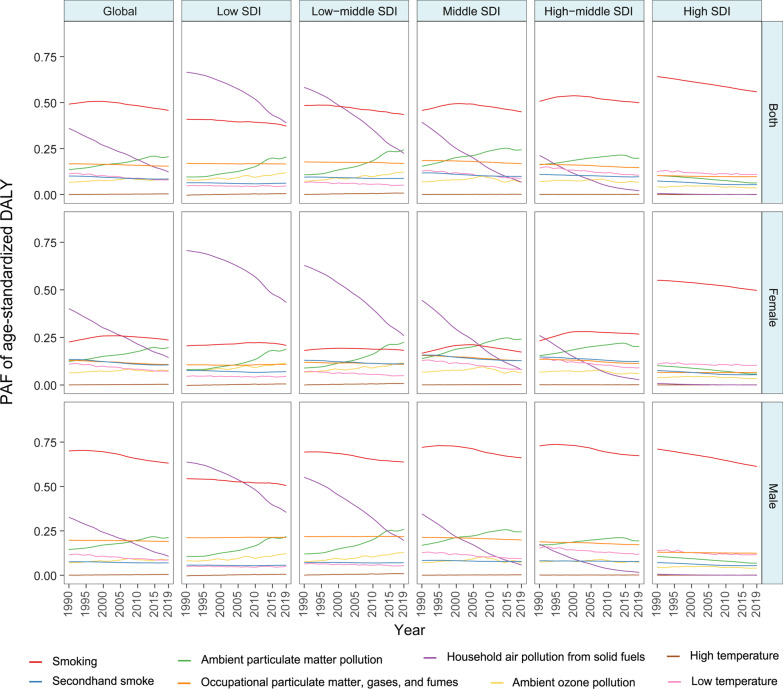
Contributions of 8 main risk factors to the PAF of age-standardized DALY due to chronic obstructive pulmonary disease by different SDI quintiles and sex from 1990 to 2019. *PAF* population attributable fraction, *DALY* disability-adjusted life year, *SDI* socio-demographic index

As shown in Fig. 2, globally, the leading risk factors in 2019 were smoking and exposure to ambient particulate matter pollution, occupational particles, and household air pollution. Although the age-standardized SEV of smoking declined in later years (Additional file 2: Fig. S1), smoking remained the top risk factor for nearly 30 years, especially in males, with the PAFs of age-standardized DALY, YLL, YLD, and death being much higher than those of the other risk factors globally (Fig. 2, Additional file 2: Figs. S2–S4). In contrast, as the age-standardized SEV of household air pollution declined, its PAFs of age-standardized DALY, YLL, YLD, and death decreased significantly (Fig. 2, Additional file 2: Figs. S2–S4). It is worth noting that in low- and low-middle-SDI regions, females were more likely to develop COPD attributable to household air pollution. The PAFs of age-standardized DALY, YLL, YLD, and death attributable to ambient particulate matter and ozone presented adverse increasing trends. Moreover, ambient particulate matter pollution became the most important risk factor among females in the middle-SDI region due to increased exposure worldwide.

**Fig. 3:**
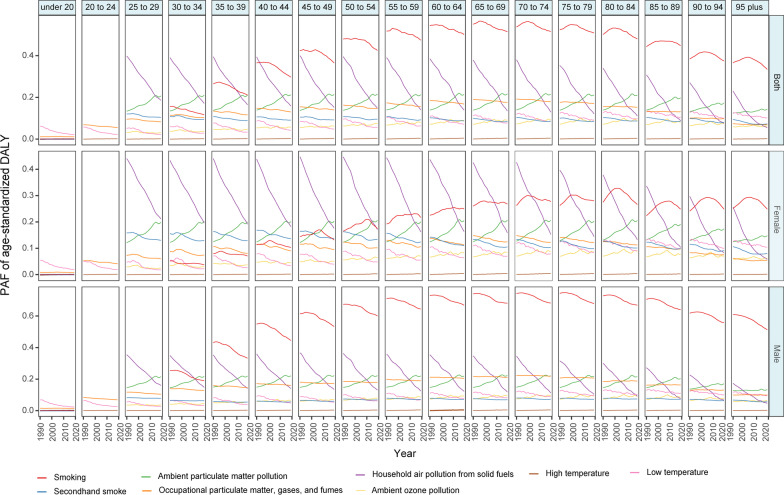
Contributions of 8 main risk factors to the PAF of age-standardized DALY due to chronic obstructive pulmonary disease by different age and sex from 1990 to 2019. *PAF* population attributable fraction, *DALY* disability-adjusted life year

The age- and sex-specific PAFs of DALYs attributable to each risk factor during the past 30 years were also highly variable (Fig. 3). In 2019, smoking was the top risk factor for COPD DALYs in the group aged more than 35 years. The contribution to the DALYs increased with age, peaked in the 65–74 years age group, and subsequently declined with age in the general population. A similar pattern was observed in the male population; meanwhile, smoking had an even higher contribution to COPD for DALYs in all age groups. For females, household air pollution and ambient particulate matter exposure were the dominant risk factors for COPD DALYs in the group aged less than 50 years. Smoking was the dominant risk factor among people aged 55 years and over. In summary, there was a sex disparity in the major risk factors among the population younger than 50, while in the elderly population, smoking imposed the heaviest burden on both males and females in 2019.

### Disease burden attributed to each risk factor

#### Smoking

**Fig. 4:**
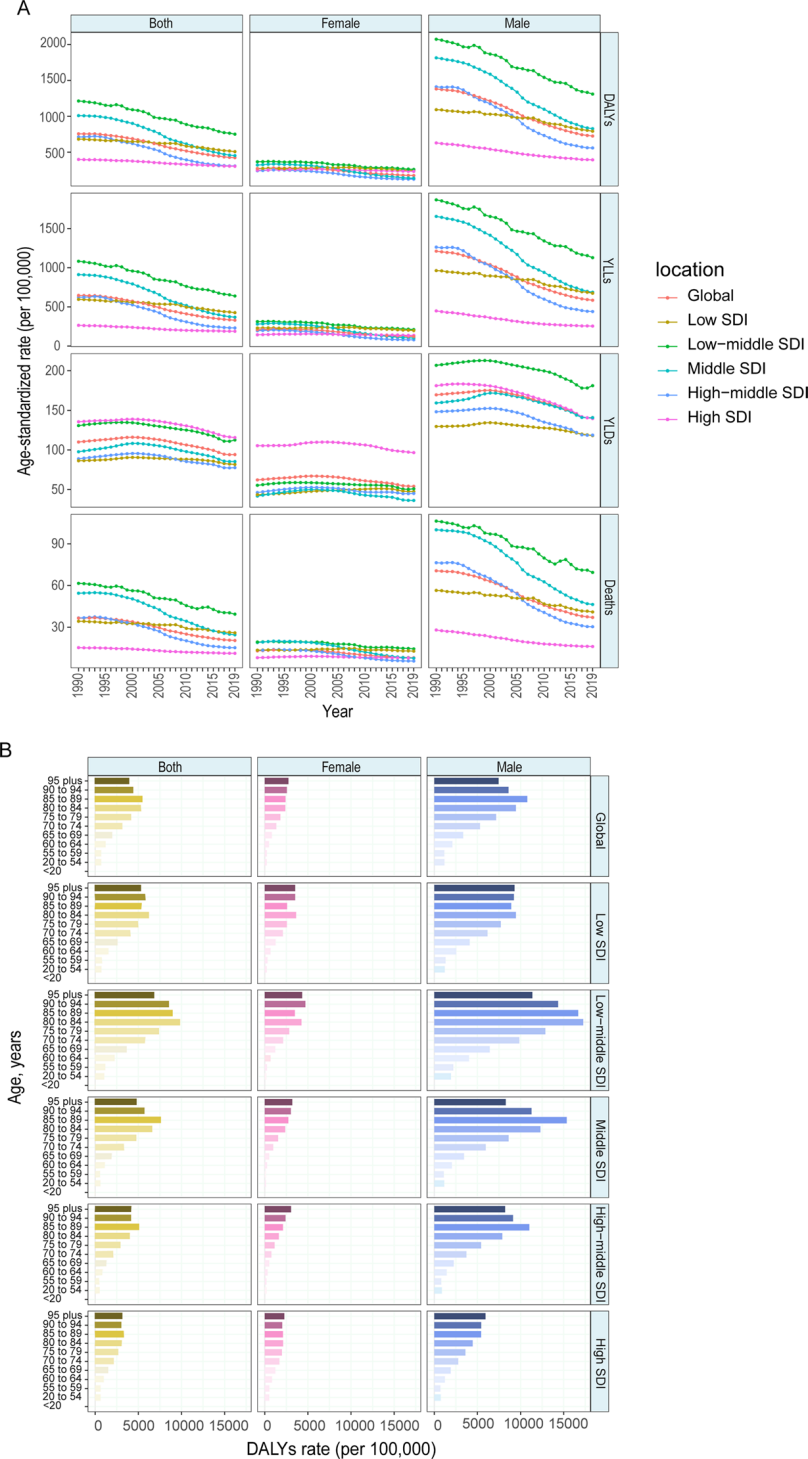

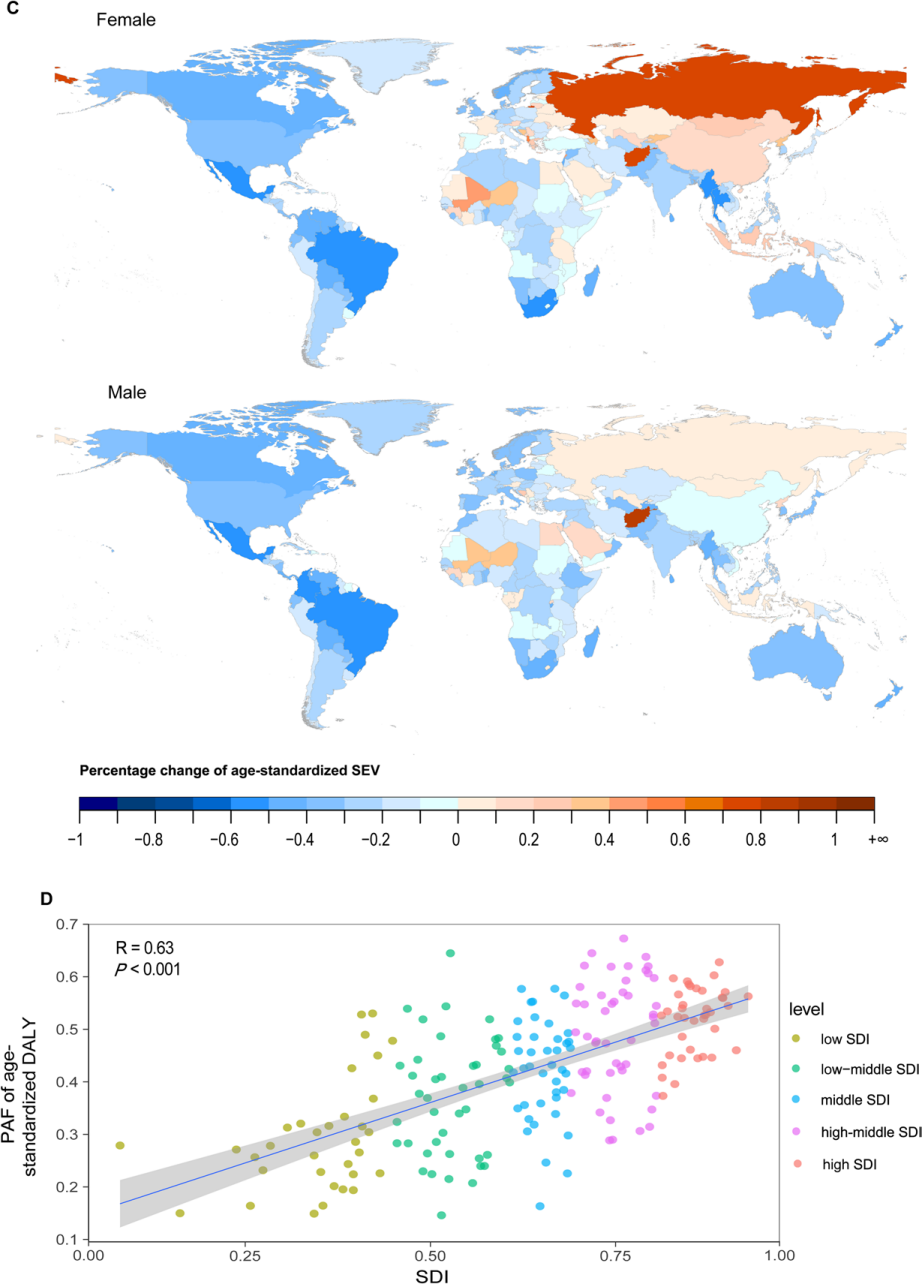
The global burden of COPD attributable to smoking over the past 30 years. **A** Age-standardized rates of DALY, YLL, YLD and death attributable to smoking, 1990–2019. **B** Age-specific DALY by SDI quintiles and sex in 2019. **C** Percentage change of age-standardized SEV of smoking for female and male in 204 countries from 1990 to 2019. **D** Relationship between PAF of age-standardized DALY and SDI in 2019. *COPD* chronic obstructive pulmonary disease, *DALY* disability-adjusted life year, *YLL* year of life lost, *YLD* year lived with disability, *SDI* socio-demographic index, *SEV* summary exposure value, *PAF* population attributable fraction

In 2019, the age-standardized DALY, YLL, and death rates of COPD attributable to smoking were the highest in the low-middle-SDI region and the lowest in the high-SDI region (Fig. 4A). The YLD rate in females was the highest in the high-SDI region. Almost all SDI regions presented declining trends of DALY, YLL, and death rates, with steeper slopes in the middle-SDI (DALY rate: EAPC − 3.95 [− 4.25–− 3.65]) and high-middle-SDI regions (DALY rate: EAPC − 4.65 [− 5.14–− 4.16]) from 2000 to 2009 (Fig. 4A, Table 1). Nevertheless, the YLD rate in each region was generally stable and even exhibited a slight increasing trend in the low-middle-SDI region in later years. These changes in disease burden were related to changes in smoking rates in certain countries and territories. Exposure in females in Afghanistan, the Russian Federation, and Albania increased, while exposure in females in Mexico, Myanmar, South Africa, Thailand, and Brazil decreased. Similarly, males in Mexico, Brazil, and South Africa had the most significantly decreased rates of exposure to smoking from 1990 to 2019 (Fig. 4C).

In all the age groups and SDI regions, the burden of disease caused by smoking was higher in males than in females in 2019. The global DALY rate of COPD attributable to smoking increased with age, peaking in those aged 85–89 and then decreasing (Fig. 4B). The PAF of age-standardized DALY attributable to smoking had a significantly positive linear correlation with the SDI (R = 0.63, *P* < 0.001) (Fig. 4D).

#### Household air pollution from solid fuels

**Fig. 5:**
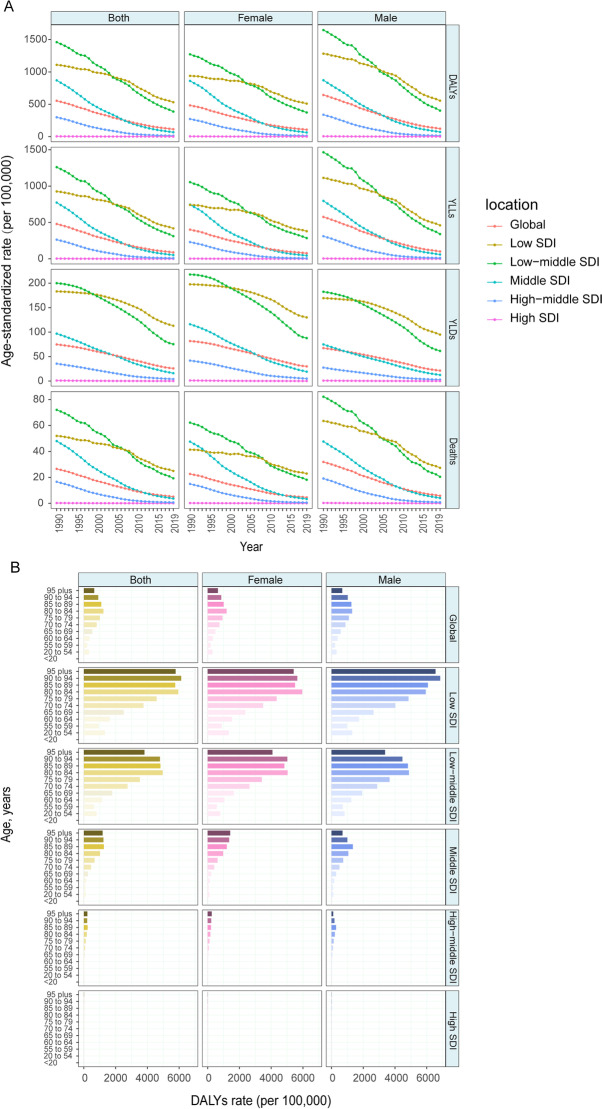

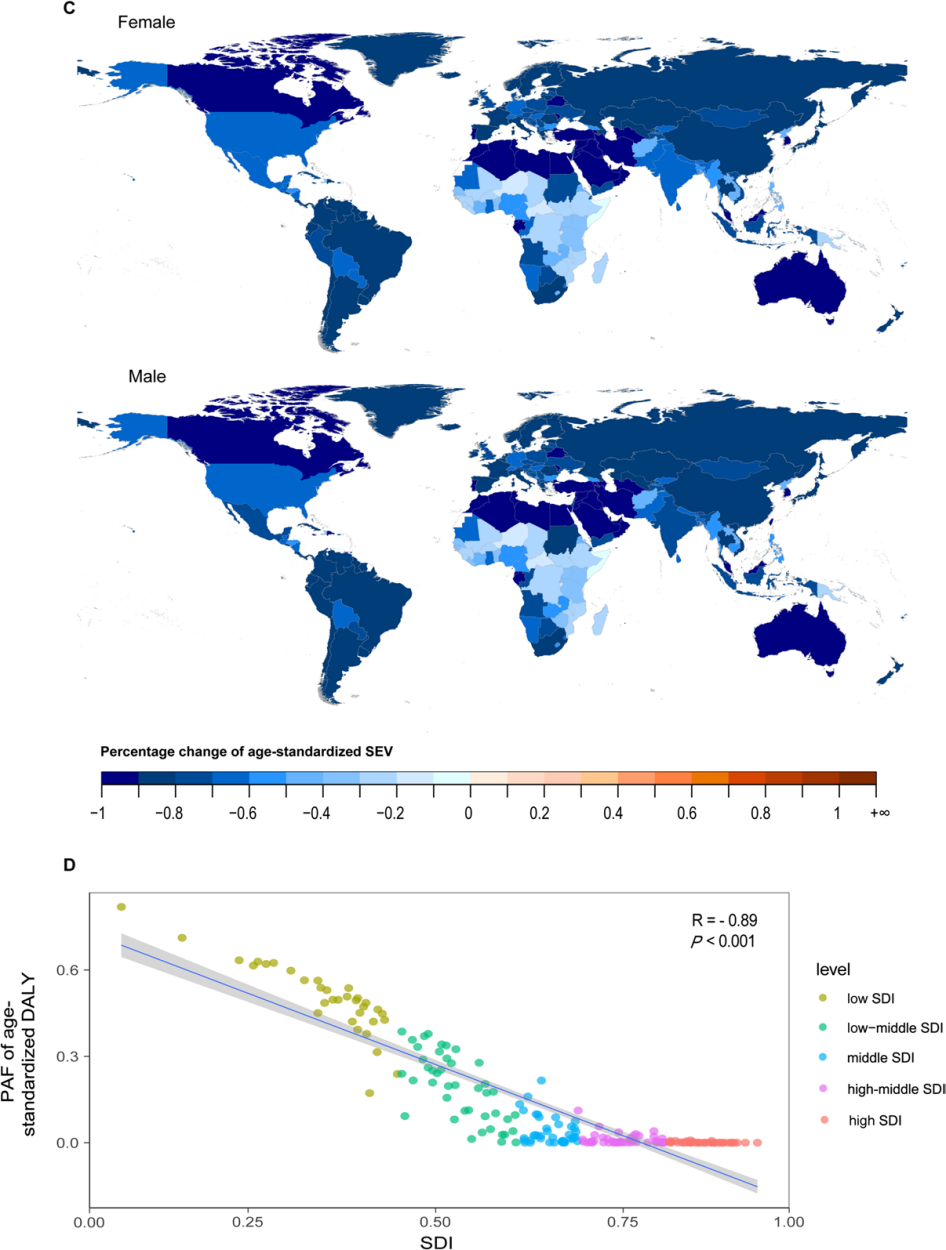
The global burden of COPD attributable to household air pollution over the past 30 years. **A** Age-standardized rates of DALY, YLL, YLD and death attributable to household air pollution, 1990–2019. **B** Age-specific DALY by SDI quintiles and sex in 2019. **C** Percentage change of age-standardized SEV of household air pollution for female and male in 204 countries from 1990 to 2019. **D** Relationship between PAF of age-standardized DALY and SDI in 2019. *COPD* chronic obstructive pulmonary disease, *DALY* disability-adjusted life year, *YLL* year of life lost, *YLD* year lived with disability, *SDI* socio-demographic index, *SEV* summary exposure value, *PAF* population attributable fraction

In 2019, the age-standardized DALY rate attributable to household air pollution was the highest in the low-SDI quintile (531.76 [343.18–744.87]), at more than 2,500-fold that in the high-SDI quintile (0.20 [0.05–0.54]) (Additional file 1: Table S1). Almost all the SDI regions showed declining DALY, YLL, YLD, and death rate trends (Fig. 5A). The largest reduction in the age-standardized DALY rate attributable to household air pollution occurred in the low-middle-SDI region and middle-SDI region. The COPD age-standardized DALY, YLL, YLD, and death rates attributable to household air pollution in 2019 were similar between males and females but slightly higher in males, except YLD (Fig. 5A, Additional file 1: Table S1). The DALY rate was the highest in those aged 80–84 years in the low-SDI region, while the burden was mild in those aged less than 55 years (Fig. 5B).

Furthermore, the PAF of age-standardized DALY attributable to household air pollution had a significantly negative linear correlation with the SDI, and the correlation was marked (R = − 0.89, P < 0.001). (Fig. 5D).

#### Ambient particulate matter pollution

**Fig. 6:**
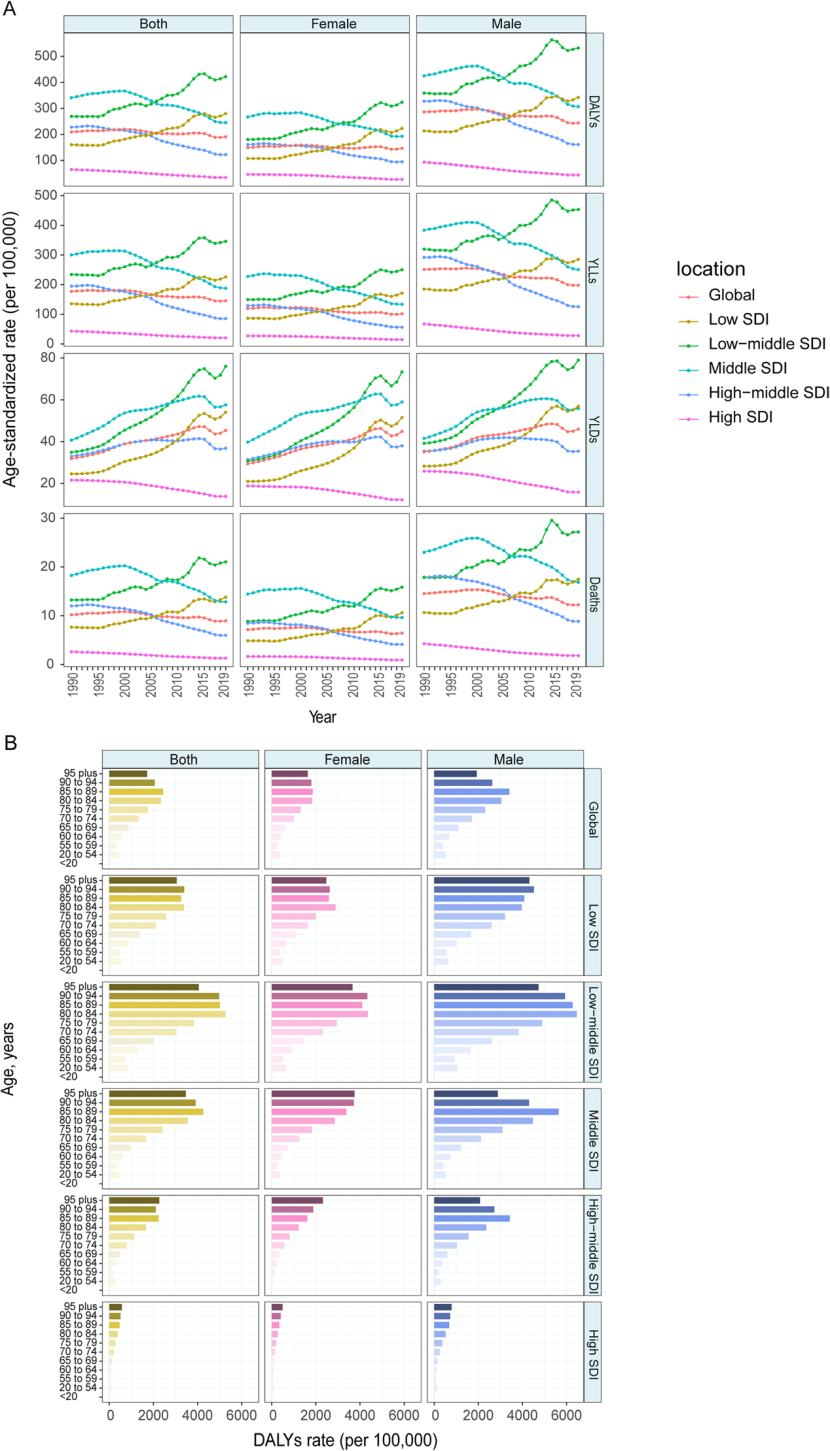

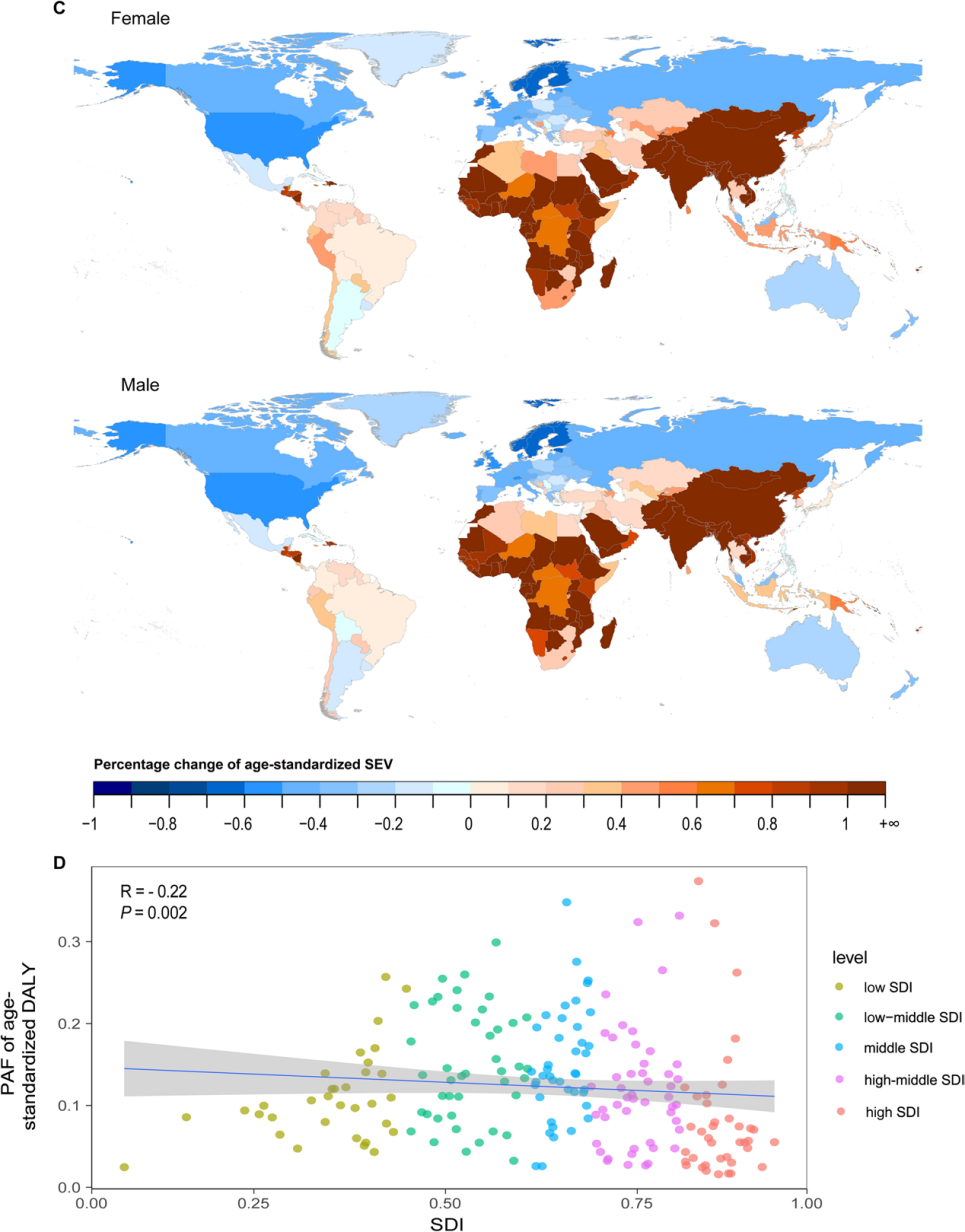
The global burden of COPD attributable to ambient particulate matter pollution over the past 30 years. **A** Age-standardized rates of DALY, YLL, YLD and death attributable to ambient particulate matter pollution, 1990 to 2019. **B** Age-specific DALY by SDI quintiles and sex in 2019. **C** Percentage change of age-standardized SEV of ambient particulate matter pollution for female and male in 204 countries from 1990 to 2019. **D** Relationship between PAF of age-standardized DALY and SDI in 2019. *COPD* chronic obstructive pulmonary disease, *DALY* disability-adjusted life year, *YLL* year of life lost, *YLD* year lived with disability, *SDI* socio-demographic index, *SEV* summary exposure value, *PAF* population attributable fraction

The disease burden of COPD attributable to ambient particulate matter pollution presented an increasing trend in the low-middle-SDI (DALY rate in 2010–2019: EPAC 1.57 [0.36–2.79]) and low-SDI quintiles (DALY rate in 2010–2019: EAPC 1.94 [0.70–3.20]) (Fig. 6A, Table 1). Conversely, the other regions showed declining burden trends, with the steepest declining slope in the high-middle-SDI region.

Additionally, in 2019, the age-standardized DALY rate in males (244.20 [194.51–300.73]) was almost 170% that in females (146.42 [112.57–184.47]) worldwide (Additional file 1: Table S1). Similarly, the global burden of COPD attributable to ambient particulate matter pollution increased with age (Fig. 6B). The PAF of age-standardized DALYs attributable to ambient particulate matter pollution had a negative linear correlation with the SDI, but the correlation was weak (R = − 0.22, P = 0.002) (Fig. 6D).

#### Occupational particulate matter, gas, and fume exposure

The age-standardized DALY, YLL, and death rates of COPD attributable to occupational particles declined in all SDI regions, but the YLD rate presented a slightly increasing trend in later years (Additional file 2: Fig. S5A). Furthermore, among all SDI regions, the greatest decline of DALY and death rates attributable to occupational particles from 1990 to 2019 was observed in the middle- and high-middle-SDI region (Additional file 2: Fig. S5A).

The COPD burden attributable to occupational particles were substantially higher in males than in females in all SDI regions (Additional file 2: Fig. S5A, B). In 2019, the global age-standardized DALY rate in males (218.10 [168.66–267.97]) was more than twice that in females (80.29 [60.92–100.38]) (Additional file 1: Table S1). Nevertheless, in some countries and territories, the age-standardized SEV of occupational particles in female showed an obvious increase. (Additional file 2: Fig. S5C). The PAF of age-standardized DALY attributable to occupational particles had a significantly negative linear correlation with the SDI (R = − 0.70, P < 0.001) (Additional file 2: Fig. S5D).

#### Secondhand smoke

Similar to that of occupational particles, the DALY, YLL, and death rates of COPD attributable to secondhand smoke steadily decreased in all SDI regions, but YLD rate showed a slightly increasing trend in later years (Additional file 2: Fig. S6A). The age-standardized SEV of secondhand smoke was considerable in the female group (Additional file 2: Fig. S1). Moreover, the DALY, YLL, and death rates of COPD attributable to secondhand smoke were similar between sexes. The YLD was higher in females than in males (Additional file 2: Fig. S6A). The PAF of age-standardized DALY attributable to secondhand smoke had a positive linear correlation with the SDI, but the correlation was weak (R = 0.21, P = 0.003) (Additional file 2: Fig. S6D).

#### Ambient ozone pollution

Compared with those in 1990, the age-standardized DALY rates in 2019 in the low-SDI (162.71 [78.42–250.27]) and low-middle-SDI regions (212.88 [103.94–332.03]) were higher (Additional file 2: Fig. S7A, Additional file 1: Table S1). The other regions had decreasing burden trends, and the rates of decline in the past decade were higher than those from 2000 to 2009 (Additional file 2: Fig. S7A, Table 1). The COPD burden attributable to ambient ozone pollution were obviously greater in males than in females. The PAF of age-standardized DALY attributable to ambient ozone pollution had a negative linear correlation with the SDI, although the correlation was weak (R = − 0.17, P = 0.014) (Additional file 2: Fig. S7D).

#### High temperature and low temperature

The burdens of disease caused by exposure to non-optimal temperatures were relatively low and certainly had regional limitations. The high temperature-attributable disease burden increased in general, especially in the low-SDI and low-middle-SDI regions, and the low-middle-SDI quintile had the greatest burden (Additional file 2: Fig. S8A). The disease burden attributable to low temperature had a declining trend, and the greatest burden was observed in the middle-SDI quintile (Additional file 2: Fig. S9A). Overall, the burden of disease attributable to exposure to non-optimal temperature was higher in males than in females, and the load also increased with age (Additional file 2: Fig. S9A, B).

Furthermore, the PAF of age-standardized DALY attributable to exposure to high temperature had a mildly negative linear correlation with the SDI (R = − 0.22, p = 0.001), while the PAF of age-standardized DALY attributable to exposure to low temperature had a mildly positive linear correlation with the SDI (R = 0.39, P < 0.001) (Additional file 2: Fig. S8–9D).

## Discussion

Previous GBD 2017 studies made a comprehensive compilation of prevalence, deaths, and DALYs for all chronic respiratory diseases (including COPD) worldwide over an extended period, and a broad overview of the risk factors for chronic respiratory diseases [2, 3]. And notably, DALY and death of COPD attributable to risk factors accounted for a significant proportion of the overall COPD burden, suggesting that prevention and control of the disease burden of COPD based on risk factors will be necessary and significant. However, the effect of risk factors on COPD burden varies with different risk factors, regions, sexes and ages, which need a more granular analysis. Thus, for more individualized prevention and control policies, we first revealed the comprehensive pattern and trend of disease burden (including DALY, YLL, YLD, death) for COPD attributable to each risk factor by different SDI regions, sexes and age groups. Our study found: (i) In 2019, smoking is the most important risk factor for male in each region. For female, the most important risk factor was household air pollution in low- and low-middle-SDI regions, was ambient particulate matter in middle-SDI-region, and was smoking in high-middle- and high-SDI regions. (ii) The COPD burden attributable to ambient particulate matter, ozone, and high-temperature exposure in low- and low-middle-SDI regions present undesirable increasing trends. (iii) Effects of smoking, ambient particulate matter, ozone, occupational particles, and low and high temperature on the COPD disease burden were obviously greater in male than in female, although the SEVs of some of these risk factors were similar between male and female.

From 1990 to 2019, the age-standardized DALY and death rates attributable to each risk factor except ambient particulate matter, ozone, and high temperature presented steadily declining trends in all SDI regions. Nevertheless, efforts to decrease the age-standardized YLD rate attributable to each risk factor have not been as effective as those to decrease the age-standardized death rate, suggesting that improving quality of life should be emphasized. Moreover, in terms of SDI region, our analysis revealed that in 2019, the age-standardized DALY and death rates of COPD attributable to each risk factor except household air pollution and low temperature were highest in the low-middle-SDI region. Surprisingly, the age-standardized SEVs of these risk factors in the low-middle-SDI region were generally either similar to those in other regions or ranked in the middle, which was inconsistent with the disease burden trends. Some possible explanations for this unexpected observation as follows: (i) the risk of developing COPD is ethnic-dependent, and ethnic differences are present across various regions; and (ii) compared with the low-middle-SDI region, the low-SDI region had a lower COPD detection rate, while higher-SDI regions possessed more advanced medical technology and more abundant medical resources.

Sex differences in the COPD DALY and death rates also existed in every subset stratified by risk factor. For females, the largest PAF of age-standardized DALY attributable to risk factors varied among the different SDI regions and ages. But for males, smoking was the absolute top risk factor. Interestingly, as Table S1 showed, the age-standardized DALY rate attributable to each risk factor was higher in males than in females and was relatively obvious for smoking and ambient particulate matter, ozone, occupational particles, and low and high temperature exposure. For smoking and occupational particles exposure, this variation could be partially explained by the variations in the SEVs between the sexes. However, for ambient particulate matter, ozone, and low and high temperature exposure showed in Additional file 2: Figure S1, there were almost no differences in the age-standardized SEVs between males and females. Air pollution (including ambient particulate matter and ozone) is an important risk factor for COPD. But cumulative evidences suggested that women appear to be more sensitive to the effects of air pollution and smoking, because of their smaller airways (which means proportionately greater exposure), higher expression of genes involved in cytochrome P450 regulation in female, and hormonally mediated differences in pollution metabolism, etc. [13–15] This does not seem to support the higher COPD burden on men. On the other hand, male patients with COPD have worse survival than female patients might explain the higher disease burden of COPD to some extent [16–18]. Possible support for this is based on phenotypic differences between men and women in COPD [13, 19]. Chronic bronchitis is more common in women and emphysema is more common in men, the latter showing a faster decline in lung function and higher mortality. Additionally, for non-optimal temperature (including low and high temperature), it has been associated with elevated mortality risk [20, 21]. The impact of high temperature might be the alteration of fluid and electrolytic balance in COPD patients [21]. Cold weather might increase airway inflammation (TNF -α, leukotrienes, prostaglandins, etc.) [22–24]. Nevertheless, little is known about whether their effects differ between sexes, which need to be further explored. Therefore, based on the available evidence, the differences in disease burden caused by temperature may also be mainly due to the poorer survival of men. But alternative explanations should not be excluded.

Smoking, a key driver of COPD progression and the only behavioral risk that is controllable, is a vitally important risk factor that should be considered. It has been reported that exposure to cigarette smoke causes the destruction of the extracellular matrix, a shortage in blood supply, and the death of epithelial cells in the lungs. [25, 26] Herein, a strong correlation between smoking and COPD was confirmed once again; although smoking had the second-lowest global exposure rate compared to other risk factors, the global PAF of age-standardized DALY attributable to smoking was far larger than those of other risk factors. In addition, unlike the PAFs of other risk factors, the PAF of DALYs attributable to smoking showed remarkable heterogeneity among teenagers, middle-aged and older people. This may reflect the chronic additive effects of smoking, which are far greater than those of other risk factors. Many strategies for the control of smoking, such as economic, cultural, media-based, and family functioning measures, have been suggested in previous studies [27–30]. As the age-standardized DALY rate attributable to smoking had the steepest decline in the slope in low- and low-middle-SDI regions between 2000 and 2009, we should explore additional practicable and valid ways to generalize smoking prevention measures. Notably, as COPD is a chronic disease, there is a certain lag from the implementation of control measures to the corresponding effect.

An extremely obvious negative correlation was observed between the PAF of age-standardized DALY attributable to household air pollution and the SDI. With the development of society, the age-standardized DALY, YLL, YLD, and death rates of COPD attributable to household air pollution showed a steady downward trend but were still higher in low- and low-middle-SDI regions than in other regions. In resource-limited settings, solid fuels such as wood and cow dung, which are easily accessible, are used as cooking fuel in less-developed countries [31]. Poor ventilation and longer exposure durations also contribute to the increased rates [32]. Therefore, the COPD burden due to household air pollution in low- and low-middle-SDI regions remains a significant concern and requires more attention.

Although the age-standardized DALY, YLL, YLD, and death rates of COPD attributable to ambient particulate matter were relatively stable at the global level, the age-standardized SEVs of and COPD burdens attributable to ambient particulate matter and ozone exposure presented undesirable increasing trends in the low- and low-middle-SDI regions, suggesting that a control strategy consisting of a series of specific measures should be designed. Public health policy- and decision-makers should prioritize the design and implementation of effective ad hoc policies to address ambient particulate matter and ozone pollution.

Unlike smoking, the attributable age-standardized DALY rate and SEV of which is similar between the high-middle-SDI region and high-SDI region, the attributable age-standardized DALY rate and SEV of secondhand smoke exposure were lower in the high-SDI region than in the high-middle-SDI region. Greater limitations regarding smoking in public places may explain this finding to some extent, which is supported by an increasing body of evidence [33–36]. In addition, the age-standardized DALY and death rates caused by exposure to occupational dust was generally higher in men than in women since men have higher exposure to hazardous. However, some regions or countries with significant increases in occupational particles exposure for women also need attention. Different regions may be associated with low temperatures or high temperatures. Interestingly, relatively consistent and obvious trends of COPD burdens attributable to low and high temperatures were observed over the entire study period (the burden attributable to exposure to high temperature increased, whereas the burden attributable to low temperature decreased). This result may be influenced by global warming.

## Conclusions

In summary, we need individualized measures for different high-risk groups according to their different pattern of COPD burden attributable to risk factors (e.g., the most important risk factors vary for women in different SDI regions). The DALY and death rates of COPD attributable to each risk factor except household air pollution and low temperature were the highest in the low-middle-SDI region. Notably, the undesirable increases in the COPD burdens attributable to ambient particulate matter, ozone, and high-temperature exposure in the low-middle- and low-SDI regions urgently need more attention and the implementation of relevant policies. Additionally, the observation that COPD burden attributable to ambient particulate matter, ozone, and low and high-temperature exposure is greater in males than in females, which couldn't be explained by the difference in SEV. Biological differences between male and female in COPD and its risk factors need to be further researched.

## Limitations

Although this GBD study fills a gap of distributions and trends of the disease burden of COPD attributable to each risk factor, several limitations should be noted. Common deficiencies, such as the use of data based on information derived from samples not necessarily representative of the whole country/territory under study, have been explained exhaustively in many previously published GBD studies [1, 2, 4, 37]. In addition, different diagnostic thresholds for airway obstruction (expiratory volume in one second/forced vital capacity < 0.70, or the lower limit of normal) may influence the diagnosis rate of COPD [38]. Moreover, the issue of confounding between air pollution and smoking, which has not been addressed, inevitably produces deviation.

## Supplementary Information

**Additional file 1: Table S1.** The age-standardized DALY rate of COPD attributable to risk factors across different SDI regions, in 2019. **Table S2.** The temporal trends of age-standardized YLL rate attributed to risk factors across different SDI regions, 1990–2019. **Table S3.** The temporal trends of age-standardized YLD rate attributed to risk factors across different SDI regions, 1990–2019. **Table S4.** The temporal trends of age-standardized death rate attributed to risk factors across different SDI regions, 1990–2019.

**Additional file 2: Figure S1.** Age-standardized rate of SEV of 8 main risk factors by SDI quintiles and sex from 1990 to 2019. **Figure S2.** Contributions of 8 main risk factors to the PAF of age-standardized death due to chronic obstructive pulmonary disease by different SDI quintiles and sexes from 1990 to 2019. **Figure S3.** Contributions of 8 main risk factors to the PAF of age-standardized YLD due to chronic obstructive pulmonary disease by different SDI quintiles and sexes from 1990 to 2019. **Figure S4.** Contributions of 8 main risk factors to the PAF of age-standardized YLL due to chronic obstructive pulmonary disease by different SDI quintiles and sexes from 1990 to 2019. **Figure S5.** The global burden of COPD attributable to occupational particles over the past 30 years. **Figure S6.** The global burden of COPD attributable to secondhand smoke over the past 30 years. **Figure S7.** The global burden of COPD attributable to ambient ozone pollution over the past 30 years. **Figure S8.** The global burden of COPD attributable to high temperature over the past 30 years. **Figure S9.** The global burden of COPD attributable to low temperature over the past 30 years.

## Data Availability

The datasets generated and/or analysed during the current study are available in the [Global Health Data Exchange GBD Results Tool] repository, [http://ghdx.healthdata.org/gbd-results-tool].

## Abbreviations

ASR: Age-standardized rate
CI: Confidence interval
COPD: Chronic obstructive pulmonary disease
DALY: Disability-adjusted life year
EAPC: Estimated annual percentage change
GBD: Global Burden of Disease
PAF: Population attributable fraction
SDI: Socio-demographic index
SEV: Summary exposure value
YLD: Year lived with disability
YLL: Year of life lost
