# Supplementary material for: Distributions and trends of the global burden of COPD attributable to risk factors by SDI, age, and sex from 1990 to 2019: a systematic analysis of GBD 2019 data
Source: Respir Res. 2022 Apr 11;23:90. doi: 10.1186/s12931-022-02011-y (PMC8996417; doi:10.1186/s12931-022-02011-y)
Supplement: Supplementary file 1 — Additional file 1: Table S1. The age-standardized DALY rate of COPD attributable to risk factors across different SDI regions, in 2019. Table S2. The temporal trends of age-standardized YLL rate attributed to risk factors across different SDI regions, 1990–2019. Table S3. The temporal trends of age-standardized YLD rate attributed to risk factors across different SDI regions, 1990–2019. Table S4. The temporal trends of age-standardized death rate attributed to risk factors across different SDI regions, 1990–2019. [file 12931_2022_2011_MOESM1_ESM.docx]

**Table S1. The age-standardized DALY rate of COPD attributable to risk factors across different SDI regions, in 2019.**

|  |  | **global** | **low SDI** | **low-middle SDI** | **middle SDI** | **high-middle SDI** | **high SDI** |
| --- | --- | --- | --- | --- | --- | --- | --- |
| **All risk factors** | **Both** | 701.81 (636.70 to 764.09) | 1130.36 (966.49 to 1283.22) | 1389.36 (1188.59 to 1566.23) | 754.89 (678.02 to 843.92) | 456.47 (412.67 to 528.56) | 376.21 (344.45 to 405.81) |
|  | **Female** | 492.07 (419.41 to 559.29) | 924.51 (710.78 to 1123.54) | 1029.78 (797.79 to 1238.12) | 499.98 (417.70 to 588.99) | 287.75 (247.65 to 365.25) | 297.86 (248.88 to 327.28) |
|  | **Male** | 957.42 (866.04 to 1044.63) | 1353.12 (1142.59 to 1553.75) | 1795.25 (1528.45 to 2048.23) | 1056.08 (936.69 to 1185.14) | 688.62 (618.61 to 786.67) | 476.36 (435.27 to 518.29) |
| **Smoking** | **Both** | 423.98 (380.20 to 465.74) | 508.09 (428.41 to 583.77) | 751.29 (625.15 to 853.05) | 453.18 (400.37 to 508.24) | 308.54 (276.09 to 350.34) | 303.69 (274.10 to 334.29) |
|  | **Female** | 175.15 (148.26 to 200.20) | 244.65 (183.57 to 303.70) | 260.91 (192.10 to 325.84) | 137.91 (111.71 to 165.44) | 124.07 (104.47 to 154.88) | 232.68 (191.11 to 260.33) |
|  | **Male** | 725.41 (648.28 to 796.37) | 792.13 (659.87 to 917.71) | 1309.46 (1100.84 to 1501.13) | 827.89 (729.80 to 935.74) | 559.14 (499.18 to 633.49) | 393.94 (352.19 to 433.56) |
| **Household air pollution** | **Both** | 114.22 (69.75 to 172.39) | 531.76 (343.18 to 744.87) | 387.18 (227.59 to 594.75) | 67.79 (32.79 to 124.20) | 13.06 (5.19 to 27.92) | 0.20 (0.05 to 0.54) |
|  | **Female** | 107.32 (66.83 to 159.89) | 509.85 (323.58 to 705.04) | 373.00 (219.14 to 565.92) | 63.96 (31.24 to 114.80) | 12.68 (5.14 to 26.71) | 0.19 (0.05 to 0.53) |
|  | **Male** | 122.54 (71.31 to 193.60) | 555.57 (330.10 to 820.23) | 401.83 (225.17 to 641.73) | 71.74 (32.33 to 136.82) | 13.67 (4.95 to 30.18) | 0.21 (0.05 to 0.60) |

(Table S1 continues to next page)

|  |  | **global** | **low SDI** | **low-middle SDI** | **middle SDI** | **high-middle SDI** | **high SDI** |
| --- | --- | --- | --- | --- | --- | --- | --- |
| **Occupational particles** | **Both** | 143.02 (118.56 to 168.69) | 227.14 (184.57 to 268.67) | 292.95 (236.01 to 349.52) | 169.64 (139.42 to 205.04) | 90.97 (74.26 to 113.17) | 52.97 (43.48 to 62.87) |
|  | **Female** | 80.29 (60.92 to 100.38) | 127.22 (91.79 to 167.54) | 155.96 (110.00 to 202.86) | 102.26 (74.27 to 132.19) | 52.81 (39.15 to 72.23) | 30.89 (23.29 to 39.29) |
|  | **Male** | 218.10 (168.66 to 267.97) | 334.63 (253.96 to 414.97) | 447.62 (340.85 to 561.39) | 248.86 (191.85 to 310.11) | 142.86 (109.54 to 183.35) | 79.85 (60.73 to 101.54) |
| **Ambient particulate matter** | **Both** | 190.79 (153.52 to 234.76) | 280.13 (197.71 to 372.36) | 422.01 (319.15 to 533.35) | 245.28 (196.83 to 305.61) | 122.22 (93.83 to 155.20) | 34.41 (21.58 to 50.38) |
|  | **Female** | 146.42 (112.57 to 184.47) | 222.92 (146.83 to 316.88) | 323.71 (223.52 to 427.27) | 192.83 (148.72 to 246.54) | 94.45 (70.84 to 128.40) | 26.90 (16.32 to 40.15) |
|  | **Male** | 244.20 (194.51 to 300.73) | 342.33 (237.31 to 459.17) | 532.58 (398.13 to 683.25) | 306.80 (242.55 to 377.05) | 160.91 (124.32 to 206.32) | 43.80 (28.04 to 63.54) |
| **Ambient ozone pollution** | **Both** | 77.04 (37.03 to 119.52) | 162.71 (78.42 to 250.27) | 212.88 (103.94 to 332.03) | 69.55 (32.53 to 109.80) | 41.74 (19.79 to 66.14) | 20.06 (8.83 to 33.33) |
|  | **Female** | 57.35 (26.95 to 90.39) | 136.00 (64.58 to 218.37) | 166.66 (77.02 to 267.51) | 51.09 (23.05 to 83.03) | 27.82 (12.58 to 45.86) | 15.59 (6.80 to 26.06) |
|  | **Male** | 100.82 (46.97 to 158.02) | 191.62 (86.93 to 296.30) | 264.69 (124.23 to 417.32) | 91.24 (41.94 to 144.65) | 61.30 (28.64 to 95.65) | 25.74 (11.29 to 42.25) |

(Table S1 continues to next page)

|  |  | **global** | **low SDI** | **low-middle SDI** | **middle SDI** | **high-middle SDI** | **high SDI** |
| --- | --- | --- | --- | --- | --- | --- | --- |
| **Secondhand smoke** | **Both** | 78.82 (39.23 to 118.67) | 85.23 (39.58 to 136.08) | 152.87 (74.32 to 236.26) | 100.22 (51.54 to 152.48) | 59.89 (30.85 to 91.17) | 29.51 (15.13 to 44.70) |
|  | **Female** | 78.58 (38.85 to 120.02) | 82.09 (39.21 to 132.85) | 160.19 (77.34 to 250.15) | 102.51 (52.04 to 157.61) | 57.74 (30.06 to 87.38) | 25.36 (12.93 to 39.10) |
|  | **Male** | 80.08 (39.90 to 123.7) | 88.9 (40.43 to 145.62) | 144.86 (71.15 to 230.67) | 97.81 (48.14 to 150.77) | 64.39 (31.66 to 100.15) | 34.83 (18.08 to 52.91) |
| **High temperature** | **Both** | 3.66 (-5.72 to 15.29) | 7.62 (-21.45 to 41.06) | 14.94 (-20.87 to 71.87) | 2.15 (-5.77 to 9.85) | 0.83 (-1.06 to 2.69) | 0.46 (-0.42 to 1.49) |
|  | **Female** | 2.72 (-4.15 to 12.14) | 6.42 (-15.97 to 33.81) | 11.40 (-16.93 to 55.23) | 1.61 (-3.70 to 6.75) | 0.57 (-0.62 to 1.94) | 0.32 (-0.26 to 0.97) |
|  | **Male** | 4.77 (-7.57 to 20.42) | 8.88 (-25.98 to 51.05) | 18.88 (-26.33 to 86.62) | 2.77 (-7.88 to 12.82) | 1.20 (-1.59 to 3.97) | 0.61 (-0.66 to 2.06) |
| **Low temperature** | **Both** | 74.43 (58.03 to 92.67) | 65.71 (31.28 to 100.59) | 90.48 (47.39 to 139.52) | 90.44 (72.26 to 113.54) | 66.00 (52.69 to 83.47) | 59.90 (48.29 to 72.14) |
|  | **Female** | 54.41 (41.59 to 69.56) | 54.09 (26.04 to 86.79) | 71.00 (38.83 to 110.55) | 66.40 (49.33 to 87.83) | 42.08 (32.79 to 60.47) | 48.67 (35.34 to 59.54) |
|  | **Male** | 99.18 (77.28 to 122.21) | 78.38 (35.24 to 121.07) | 112.07 (57.67 to 173.20) | 118.75 (93.50 to 149.28) | 99.03 (78.48 to 121.32) | 74.57 (60.13 to 89.75) |

DALY: disability-adjusted life year, SDI: socio-demographic index

**Table S2.** **The temporal trends of age-standardized YLL rate attributed to risk factors across different SDI regions, 1990 to 2019.**

|  |  | **All risk factors** | **Smoking** | **Household air pollution** | **Occupational particles** | **Ambient particulate matter** | **Ambient ozone pollution** | **Secondhand smoke** | **High temperature** | **Low temperature** |
| --- | --- | --- | --- | --- | --- | --- | --- | --- | --- | --- |
| **Global EAPC (95%CI)** | **1990-1999** | -1.76 (-2.02 to -1.51) | -1.13 (-1.44 to -0.82) | -4.38 (-4.72 to -4.04) | -1.76 (-2.01 to -1.50) | 0.18 (0.06 to 0.29) | 0.46 (0.23 to 0.70) | -2.26 (-2.65 to -1.86) | 15.04 (8.20 to 22.3) | -2.52 (-3.33 to -1.71) |
|  | **2000-2009** | -3.31 (-3.47 to -3.15) | -3.41 (-3.61 to -3.21) | -6.00 (-6.27 to -5.74) | -3.29 (-3.48 to -3.11) | -1.60 (-1.84 to -1.36) | -0.60 (-1.31 to 0.11) | -4.00 (-4.20 to -3.81) | 2.39 (-0.31 to 5.16) | -4.25 (-4.69 to -3.81) |
|  | **2010-2019** | -2.32 (-2.55 to -2.10) | -2.44 (-2.66 to -2.22) | -6.43 (-6.69 to -6.16) | -2.33 (-2.61 to -2.05) | -1.16 (-1.65 to -0.67) | -1.26 (-2.11 to -0.40) | -2.18 (-2.56 to -1.81) | 2.06 (-0.10 to 4.26) | -2.99 (-3.53 to -2.44) |
| **Low SDI EAPC (95%CI)** | **1990-1999** | -0.58 (-0.77 to -0.40) | -0.59 (-0.79 to -0.40) | -1.21 (-1.38 to -1.04) | -0.60 (-0.79 to -0.41) | 0.94 (0.20 to 1.67) | 1.07 (0.30 to 1.85) | -0.73 (-0.92 to -0.55) | -78.71 (NA to NA) | -0.55 (-1.15 to 0.06) |
|  | **2000-2009** | -0.85 (-1.10 to -0.59) | -0.79 (-1.03 to -0.55) | -2.26 (-2.59 to -1.92) | -0.61 (-0.83 to -0.38) | 2.27 (1.92 to 2.62) | 1.73 (0.56 to 2.90) | -1.25 (-1.51 to -1.00) | 20.89 (10.16 to 32.66) | -1.18 (-1.83 to -0.52) |
|  | **2010-2019** | -1.58 (-1.78 to -1.39) | -1.70 (-1.85 to -1.55) | -4.32 (-4.66 to -3.97) | -1.28 (-1.47 to -1.09) | 1.79 (0.58 to 3.02) | 1.51 (1.08 to 1.94) | -0.46 (-0.61 to -0.31) | 3.65 (-0.23 to 7.68) | -1.01 (-2.19 to 0.19) |
| **Low-middle SDI EAPC (95%CI)** | **1990-1999** | -1.28 (-1.54 to -1.02) | -1.05 (-1.33 to -0.77) | -2.75 (-3.05 to -2.45) | -1.19 (-1.47 to -0.91) | 1.02 (0.35 to 1.70) | 1.49 (0.90 to 2.08) | -1.36 (-1.58 to -1.14) | 25.14 (9.75 to 42.69) | -2.01 (-2.72 to -1.30) |
|  | **2000-2009** | -2.12 (-2.52 to -1.72) | -2.19 (-2.56 to -1.82) | -4.74 (-5.16 to -4.33) | -1.81 (-2.21 to -1.41) | 1.22 (0.58 to 1.87) | 0.55 (-0.52 to 1.63) | -2.40 (-2.74 to -2.07) | 3.07 (0.43 to 5.78) | -2.68 (-3.19 to -2.17) |
|  | **2010-2019** | -1.72 (-1.89 to -1.55) | -1.92 (-2.11 to -1.72) | -6.13 (-6.26 to -6.00) | -1.75 (-1.88 to -1.62) | 1.54 (0.31 to 2.79) | 1.35 (1.04 to 1.65) | -1.27 (-1.42 to -1.12) | 2.67 (0.34 to 5.05) | -2.77 (-3.55 to -1.98) |

(Table S2 continues to next page)

|  |  | **All risk factors** | **Smoking** | **Household air pollution** | **Occupational particles** | **Ambient particulate matter** | **Ambient ozone pollution** | **Secondhand smoke** | **High temperature** | **Low temperature** |
| --- | --- | --- | --- | --- | --- | --- | --- | --- | --- | --- |
| **Middle SDI EAPC (95%CI)** | **1990-1999** | -2.48 (-2.81 to -2.16) | -1.29 (-1.63 to -0.96) | -6.53 (-7.16 to -5.90) | -2.36 (-2.69 to -2.03) | 0.48 (0.31 to 0.66) | -0.11 (-0.50 to 0.29) | -2.95 (-3.49 to -2.41) | 7.33 (-1.99 to 17.53) | -3.14 (-3.98 to -2.29) |
|  | **2000-2009** | -4.56 (-4.83 to -4.28) | -4.42 (-4.76 to -4.08) | -9.60 (-10.32 to -8.88) | -4.56 (-4.88 to -4.23) | -2.62 (-2.96 to -2.29) | -1.09 (-1.90 to -0.28) | -5.14 (-5.42 to -4.85) | -1.04 (-5.36 to 3.47) | -5.55 (-6.07 to -5.02) |
|  | **2010-2019** | -3.83 (-4.23 to -3.43) | -3.81 (-4.18 to -3.45) | -10.56 (-10.77 to -10.35) | -3.75 (-4.18 to -3.33) | -3.32 (-3.62 to -3.02) | -5.11 (-7.04 to -3.15) | -3.63 (-4.15 to -3.11) | -0.54 (-4.38 to 3.45) | -4.34 (-5.07 to -3.61) |
| **High-middle SDI EAPC (95%CI)** | **1990-1999** | -2.62 (-3.24 to -2.00) | -1.74 (-2.46 to -1.01) | -8.37 (-9.15 to -7.58) | -2.73 (-3.32 to -2.13) | -1.12 (-1.62 to -0.63) | -1.05 (-1.79 to -0.31) | -3.05 (-3.64 to -2.46) | -0.72 (-7.97 to 7.11) | -3.18 (-4.21 to -2.14) |
|  | **2000-2009** | -5.40 (-5.95 to -4.86) | -5.43 (-6.00 to -4.85) | -13.28 (-14.39 to -12.17) | -5.66 (-6.27 to -5.04) | -4.03 (-4.62 to -3.43) | -2.88 (-3.63 to -2.12) | -5.62 (-6.20 to -5.04) | -6.21 (-9.49 to -2.81) | -5.57 (-6.20 to -4.93) |
|  | **2010-2019** | -3.24 (-3.89 to -2.60) | -3.17 (-3.80 to -2.55) | -11.07 (-11.89 to -10.24) | -3.38 (-4.10 to -2.66) | -3.90 (-4.25 to -3.55) | -3.07 (-4.60 to -1.51) | -3.43 (-4.28 to -2.57) | -0.05 (-6.22 to 6.52) | -3.62 (-4.37 to -2.85) |
| **High SDI EAPC (95%CI)** | **1990-1999** | -0.82 (-0.99 to -0.65) | -0.98 (-1.14 to -0.81) | -11.52 (-12.15 to -10.90) | -1.03 (-1.20 to -0.87) | -1.92 (-2.09 to -1.74) | 1.57 (0.89 to 2.26) | -1.82 (-2.01 to -1.64) | 5.72 (1.23 to 10.40) | -0.85 (-1.54 to -0.15) |
|  | **2000-2009** | -1.71 (-1.88 to -1.55) | -1.85 (-2.01 to -1.70) | -12.84 (-13.11 to -12.57) | -1.64 (-1.80 to -1.47) | -3.33 (-3.48 to -3.18) | -1.90 (-2.73 to -1.06) | -3.08 (-3.28 to -2.88) | 1.49 (-1.77 to 4.86) | -1.60 (-2.06 to -1.13) |
|  | **2010-2019** | -0.57 (-0.66 to -0.48) | -0.67 (-0.75 to -0.59) | -7.15 (-7.79 to -6.51) | -0.14 (-0.23 to -0.06) | -2.71 (-3.06 to -2.35) | -1.96 (-2.60 to -1.32) | -0.36 (-0.52 to -0.20) | -0.52 (-4.51 to 3.64) | -0.85 (-1.27 to -0.44) |

YLL: year of life lost, SDI: socio-demographic index, EAPC: estimated annual percentage change, CI: confidence interval

**Table S3.** **The temporal trends of age-standardized YLD rate attributed to risk factors across different SDI regions, 1990 to 2019.**

|  |  | **All risk factors** | **Smoking** | **Household air pollution** | **Occupational particles** | **Ambient particulate matter** | **Ambient ozone pollution** | **Secondhand smoke** | **High temperature** | **Low temperature** |
| --- | --- | --- | --- | --- | --- | --- | --- | --- | --- | --- |
| **Global EAPC (95%CI)** | **1990-1999** | 0.17 (0.15 to 0.19) | 0.57 (0.55 to 0.59) | -2.01 (-2.24 to -1.78) | 0.60 (0.59 to 0.61) | 1.99 (1.76 to 2.22) | - | 0.08 (-0.07 to 0.23) | - | - |
|  | **2000-2009** | -0.87 (-0.98 to -0.76) | -0.86 (-1.01 to -0.71) | -3.61 (-3.87 to -3.35) | -0.52 (-0.64 to -0.41) | 1.06 (1.00 to 1.13) | - | -1.18 (-1.27 to -1.09) | - | - |
|  | **2010-2019** | -1.39 (-1.65 to -1.12) | -1.52 (-1.71 to -1.32) | -5.30 (-5.53 to -5.06) | -0.98 (-1.22 to -0.73) | -0.02 (-0.83 to 0.79) | - | -0.77 (-1.05 to -0.49) | - | - |
| **Low SDI EAPC (95%CI)** | **1990-1999** | 0.22 (0.14 to 0.29) | 0.49 (0.39 to 0.60) | -0.34 (-0.38 to -0.29) | 0.44 (0.37 to 0.52) | 1.95 (1.25 to 2.66) | - | 0.11 (0.09 to 0.13) | - | - |
|  | **2000-2009** | -0.41 (-0.45 to -0.37) | -0.29 (-0.31 to -0.28) | -1.56 (-1.71 to -1.41) | -0.07 (-0.12 to -0.02) | 2.76 (2.41 to 3.11) | - | -0.68 (-0.7 to -0.66) | - | - |
|  | **2010-2019** | -0.99 (-1.17 to -0.81) | -0.95 (-1.13 to -0.78) | -3.20 (-3.46 to -2.95) | -0.35 (-0.47 to -0.23) | 2.60 (1.19 to 4.03) | - | 0.07 (-0.09 to 0.23) | - | - |
| **Low-middle SDI EAPC (95%CI)** | **1990-1999** | 0.00 (-0.09 to 0.08) | 0.34 (0.24 to 0.43) | -1.28 (-1.55 to -1.01) | 0.51 (0.46 to 0.57) | 2.53 (1.98 to 3.09) | - | 0.21 (0.03 to 0.39) | - | - |
|  | **2000-2009** | -0.73 (-0.75 to -0.71) | -0.73 (-0.75 to -0.71) | -3.03 (-3.22 to -2.85) | -0.20 (-0.22 to -0.18) | 2.94 (2.76 to 3.13) | - | -0.62 (-0.64 to -0.59) | - | - |
|  | **2010-2019** | -1.66 (-2.05 to -1.28) | -1.45 (-1.76 to -1.15) | -5.86 (-6.20 to -5.51) | -1.15 (-1.47 to -0.82) | 1.72 (0.56 to 2.88) | - | -0.77 (-1.17 to -0.37) | - | - |

(Table S3 continues to next page)

|  |  | **All risk factors** | **Smoking** | **Household air pollution** | **Occupational particles** | **Ambient particulate matter** | **Ambient ozone pollution** | **Secondhand smoke** | **High temperature** | **Low temperature** |
| --- | --- | --- | --- | --- | --- | --- | --- | --- | --- | --- |
| **Middle SDI EAPC (95%CI)** | **1990-1999** | -0.07 (-0.08 to -0.05) | 1.07 (1.01 to 1.13) | -3.84 (-4.12 to -3.55) | 0.33 (0.31 to 0.35) | 2.87 (2.71 to 3.03) | - | -0.04 (-0.24 to 0.16) | - | - |
|  | **2000-2009** | -1.29 (-1.40 to -1.18) | -0.97 (-1.16 to -0.77) | -5.97 (-6.53 to -5.41) | -1.00 (-1.12 to -0.88) | 0.94 (0.80 to 1.09) | - | -1.41 (-1.47 to -1.36) | - | - |
|  | **2010-2019** | -1.62 (-1.91 to -1.32) | -1.70 (-1.93 to -1.47) | -8.09 (-8.38 to -7.81) | -1.34 (-1.60 to -1.07) | -0.72 (-1.41 to -0.03) | - | -0.94 (-1.23 to -0.65) | - | - |
| **High-middle SDI EAPC (95%CI)** | **1990-1999** | 0.13 (0.10 to 0.16) | 0.78 (0.76 to 0.81) | -4.40 (-4.74 to -4.06) | 0.56 (0.54 to 0.58) | 1.66 (1.51 to 1.81) | - | 0.04 (-0.09 to 0.16) | - | - |
|  | **2000-2009** | -1.22 (-1.45 to -0.98) | -1.08 (-1.34 to -0.82) | -8.60 (-9.31 to -7.89) | -1.04 (-1.30 to -0.78) | 0.41 (0.15 to 0.67) | - | -1.33 (-1.59 to -1.06) | - | - |
|  | **2010-2019** | -1.25 (-1.55 to -0.96) | -1.21 (-1.43 to -0.99) | -8.40 (-8.60 to -8.20) | -1.13 (-1.40 to -0.87) | -1.42 (-2.26 to -0.56) | - | -1.04 (-1.31 to -0.76) | - | - |
| **High SDI EAPC (95%CI)** | **1990-1999** | 0.25 (0.22 to 0.28) | 0.23 (0.19 to 0.26) | -8.67 (-8.93 to -8.42) | 0.51 (0.47 to 0.54) | -0.45 (-0.52 to -0.38) | - | -0.56 (-0.68 to -0.44) | - | - |
|  | **2000-2009** | 1.23 (-3.68 to 6.38) | -0.61 (-0.72 to -0.50) | -10.24 (-10.42 to -10.07) | -0.36 (-0.44 to -0.28) | -1.91 (-2.04 to -1.78) | - | -2.12 (-2.18 to -2.06) | - | - |
|  | **2010-2019** | -1.31 (-1.42 to -1.19) | -1.43 (-1.54 to -1.32) | -7.09 (-7.43 to -6.76) | -0.91 (-1.02 to -0.80) | -2.55 (-2.88 to -2.23) | - | -0.95 (-1.13 to -0.76) | - | - |

YLD: year lived with disability, SDI: socio-demographic index, EAPC: estimated annual percentage change, CI: confidence interval

**Table S4.** **The temporal trends of age-standardized death rate attributed to risk factors across different SDI regions, 1990 to 2019.**

|  |  | **All risk factors** | **Smoking** | **Household air pollution** | **Occupational particles** | **Ambient particulate matter** | **Ambient ozone pollution** | **Secondhand smoke** | **High temperature** | **Low temperature** |
| --- | --- | --- | --- | --- | --- | --- | --- | --- | --- | --- |
| **Global EAPC (95%CI)** | **1990-1999** | -1.36 (-1.58 to -1.13) | -0.65 (-0.92 to -0.37) | -4.16 (-4.51 to -3.81) | -1.33 (-1.57 to -1.10) | 0.60 (0.50 to 0.70) | 0.87 (0.63 to 1.11) | -1.85 (-2.23 to -1.47) | 12.79 (6.83 to 19.09) | -1.99 (-2.76 to -1.21) |
|  | **2000-2009** | -3.02 (-3.19 to -2.86) | -3.11 (-3.32 to -2.90) | -5.98 (-6.28 to -5.68) | -3.02 (-3.21 to -2.83) | -1.37 (-1.60 to -1.15) | -0.28 (-0.99 to 0.43) | -3.71 (-3.90 to -3.53) | 2.18 (-0.49 to 4.92) | -3.76 (-4.18 to -3.34) |
|  | **2010-2019** | -2.13 (-2.35 to -1.90) | -2.26 (-2.47 to -2.05) | -6.23 (-6.43 to -6.02) | -2.09 (-2.36 to -1.83) | -1.06 (-1.59 to -0.52) | -1.23 (-2.09 to -0.37) | -2.02 (-2.41 to -1.62) | 2.48 (0.30 to 4.71) | -2.82 (-3.36 to -2.27) |
| **Low SDI EAPC (95%CI)** | **1990-1999** | -0.47 (-0.66 to -0.29) | -0.46 (-0.65 to -0.28) | -1.13 (-1.29 to -0.96) | -0.42 (-0.61 to -0.24) | 1.06 (0.31 to 1.82) | 1.19 (0.38 to 2.00) | -0.70 (-0.89 to -0.52) | -68.42 (NA to NA) | -0.41 (-1.01 to 0.19) |
|  | **2000-2009** | -0.59 (-0.88 to -0.29) | -0.59 (-0.87 to -0.32) | -2.00 (-2.36 to -1.63) | -0.33 (-0.59 to -0.07) | 2.53 (2.16 to 2.89) | 2.02 (0.81 to 3.24) | -1.07 (-1.36 to -0.78) | 18.97 (9.45 to 29.30) | -0.92 (-1.58 to -0.26) |
|  | **2010-2019** | -1.17 (-1.44 to -0.90) | -1.36 (-1.58 to -1.13) | -3.96 (-4.30 to -3.63) | -0.94 (-1.19 to -0.69) | 2.37 (1.07 to 3.67) | 2.12 (1.64 to 2.60) | -0.01 (-0.25 to 0.24) | 4.62 (0.63 to 8.77) | -0.74 (-1.97 to 0.50) |
| **Low-middle SDI EAPC (95%CI)** | **1990-1999** | -1.03 (-1.25 to -0.81) | -0.75 (-1.00 to -0.50) | -2.54 (-2.81 to -2.27) | -0.91 (-1.17 to -0.66) | 1.32 (0.68 to 1.97) | 1.69 (1.12 to 2.26) | -1.13 (-1.33 to -0.92) | 23.37 (10.15 to 38.18) | -1.74 (-2.47 to -1.01) |
|  | **2000-2009** | -1.84 (-2.25 to -1.43) | -1.89 (-2.25 to -1.52) | -4.48 (-4.94 to -4.01) | -1.53 (-1.93 to -1.12) | 1.46 (0.85 to 2.08) | 0.84 (-0.25 to 1.95) | -2.10 (-2.46 to -1.75) | 3.28 (0.69 to 5.94) | -2.33 (-2.82 to -1.83) |
|  | **2010-2019** | -1.34 (-1.75 to -0.93) | -1.53 (-1.99 to -1.06) | -5.70 (-5.95 to -5.45) | -1.36 (-1.76 to -0.97) | 1.96 (0.51 to 3.43) | 1.76 (1.40 to 2.13) | -0.97 (-1.31 to -0.62) | 3.36 (0.88 to 5.91) | -2.55 (-3.33 to -1.76) |

(Table S4 continues to next page)

|  |  | **All risk factors** | **Smoking** | **Household air pollution** | **Occupational particles** | **Ambient particulate matter** | **Ambient ozone pollution** | **Secondhand smoke** | **High temperature** | **Low temperature** |
| --- | --- | --- | --- | --- | --- | --- | --- | --- | --- | --- |
| **Middle SDI EAPC (95%CI)** | **1990-1999** | -1.89 (-2.21 to -1.57) | -0.64 (-0.97 to -0.30) | -5.88 (-6.49 to -5.26) | -1.77 (-2.09 to -1.45) | 1.12 (0.96 to 1.28) | 0.52 (0.12 to 0.92) | -2.33 (-2.85 to -1.80) | 7.76 (-1.85 to 18.30) | -2.40 (-3.25 to -1.55) |
|  | **2000-2009** | -4.15 (-4.40 to -3.90) | -4.01 (-4.32 to -3.69) | -9.16 (-9.84 to -8.47) | -4.13 (-4.43 to -3.83) | -2.18 (-2.51 to -1.85) | -0.58 (-1.39 to 0.23) | -4.69 (-4.94 to -4.44) | -0.89 (-5.37 to 3.81) | -4.99 (-5.48 to -4.51) |
|  | **2010-2019** | -3.76 (-4.16 to -3.35) | -3.77 (-4.13 to -3.40) | -10.43 (-10.62 to -10.23) | -3.63 (-4.06 to -3.21) | -3.18 (-3.52 to -2.84) | -5.23 (-7.23 to -3.19) | -3.55 (-4.08 to -3.02) | -0.36 (-4.54 to 4.00) | -4.22 (-4.96 to -3.47) |
| **High-middle SDI EAPC (95%CI)** | **1990-1999** | -2.14 (-2.63 to -1.66) | -1.13 (-1.70 to -0.56) | -7.81 (-8.55 to -7.08) | -2.22 (-2.71 to -1.73) | -0.54 (-0.91 to -0.16) | -0.49 (-1.10 to 0.13) | -2.60 (-3.10 to -2.09) | -0.22 (-7.61 to 7.76) | -2.74 (-3.65 to -1.83) |
|  | **2000-2009** | -4.88 (-5.36 to -4.39) | -4.94 (-5.47 to -4.40) | -12.97 (-13.98 to -11.94) | -5.21 (-5.76 to -4.65) | -3.49 (-4.03 to -2.94) | -2.18 (-2.89 to -1.46) | -5.15 (-5.64 to -4.66) | -5.63 (-8.99 to -2.14) | -4.95 (-5.51 to -4.38) |
|  | **2010-2019** | -3.14 (-3.77 to -2.50) | -3.12 (-3.74 to -2.50) | -10.95 (-11.77 to -10.12) | -3.20 (-3.91 to -2.48) | -3.72 (-4.08 to -3.35) | -2.84 (-4.34 to -1.31) | -3.31 (-4.16 to -2.46) | -0.03 (-6.57 to 6.96) | -3.46 (-4.21 to -2.70) |
| **High SDI EAPC (95%CI)** | **1990-1999** | -0.45 (-0.60 to -0.30) | -0.61 (-0.76 to -0.45) | -10.89 (-11.49 to -10.28) | -0.67 (-0.82 to -0.51) | -1.64 (-1.79 to -1.49) | 1.98 (1.27 to 2.70) | -1.41 (-1.59 to -1.23) | 5.88 (1.02 to 10.98) | -0.49 (-1.18 to 0.20) |
|  | **2000-2009** | -1.65 (-1.81 to -1.49) | -1.81 (-1.96 to -1.66) | -11.95 (-12.23 to -11.66) | -1.56 (-1.71 to -1.40) | -3.21 (-3.38 to -3.05) | -1.78 (-2.65 to -0.90) | -3.07 (-3.25 to -2.89) | 1.03 (-2.24 to 4.41) | -1.48 (-1.92 to -1.03) |
|  | **2010-2019** | -0.72 (-0.85 to -0.60) | -0.84 (-0.93 to -0.74) | -6.84 (-7.39 to -6.28) | -0.22 (-0.31 to -0.12) | -2.68 (-3.05 to -2.30) | -2.00 (-2.70 to -1.30) | -0.45 (-0.64 to -0.26) | -0.95 (-5.27 to 3.55) | -0.96 (-1.37 to -0.54) |

SDI: socio-demographic index, EAPC: estimated annual percentage change, CI: confidence interval

**Table S5. Countries and territories involved in each SDI region.**

| **SDI regions** | **Involved countries and territories** |
| --- | --- |
| **Low SDI** | Somalia, Niger, Chad, Burkina Faso, Mali, Central African Republic, Burundi, Mozambique, Guinea, Afghanistan, Ethiopia, Sierra Leone, Benin, Guinea-Bissau, South Sudan, Liberia, Democratic Republic of the Congo, Malawi, Senegal, Papua New Guinea, Eritrea, Madagascar, Gambia, Uganda, Solomon Islands, Cote d'Ivoire, Yemen, Togo, Nepal, United Republic of Tanzania, Rwanda, Haiti, Pakistan |
| **Low-middle SDI** | Bhutan, Comoros, Djibouti, Cambodia, Angola, Zimbabwe, Bangladesh, Vanuatu, Cameroon, Lao People's Democratic Republic, Honduras, Mauritania, Sao Tome and Principe, Zambia, Lesotho, Kenya, Timor-Leste, Nigeria, Sudan, Nicaragua, Myanmar, Cabo Verde, Guatemala, Kiribati, Tajikistan, Marshall Islands, Morocco, Ghana, Democratic People's Republic of Korea, Maldives, Bolivia (Plurinational State of), India, Congo, El Salvador, Eswatini, Micronesia (Federated States of), Palestine, Tuvalu, Dominican Republic, Kyrgyzstan, Belize, Mongolia, Venezuela (Bolivarian Republic of) |
| **Middle SDI** | Namibia, Viet Nam, Guyana, Nauru, Syrian Arab Republic, Philippines, Tokelau, Saint Vincent and the Grenadines, Uzbekistan, Colombia, Botswana, Suriname, Tonga, Paraguay, Brazil, Ecuador, Samoa, Peru, Mexico, Algeria, Gabon, Egypt, Indonesia, Fiji, Cuba, Grenada, Iran (Islamic Republic of), Saint Lucia, Turkmenistan, Iraq, Tunisia, South Africa, Costa Rica, Albania, Azerbaijan, Jamaica, Equatorial Guinea, China, Panama, Thailand, Armenia |
| **High-middle SDI** | Sri Lanka, Republic of Moldova, Uruguay, Georgia, Mauritius, Argentina, Lebanon, Libya, Niue, American Samoa, Bosnia and Herzegovina, Kazakhstan, Seychelles, Dominica, Jordan, Ukraine, Malaysia, Palau, Barbados, Antigua and Barbuda, Portugal, North Macedonia, Belarus, Saint Kitts and Nevis, Turkey, Bahrain, Trinidad and Tobago, Chile, Romania, Greenland, Bulgaria, Cook Islands, Serbia, Spain, Northern Mariana Islands, Oman, Hungary, Montenegro, Croatia, Greece, Bahamas, United States Virgin Islands, Italy, Malta, Poland, Israel, Russian Federation, Saudi Arabia |
| **High SDI** | Slovakia, Bermuda, Guam, Puerto Rico, Latvia, Brunei Darussalam, Czechia, Qatar, France, Estonia, Australia, New Zealand, Slovenia, Cyprus, Lithuania, United Kingdom, Austria, Belgium, Kuwait, Finland, United States of America, Singapore, Ireland, Taiwan (Province of China), Iceland, Japan, Sweden, Canada, Republic of Korea, United Arab Emirates, Netherlands, San Marino, Denmark, Andorra, Luxembourg, Germany, Monaco, Norway, Switzerland |

SDI: socio-demographic index
