# Supplementary figures and images for: Distributions and trends of the global burden of COPD attributable to risk factors by SDI, age, and sex from 1990 to 2019: a systematic analysis of GBD 2019 data
Source: Respir Res. 2022 Apr 11;23:90. doi: 10.1186/s12931-022-02011-y (PMC8996417; doi:10.1186/s12931-022-02011-y)

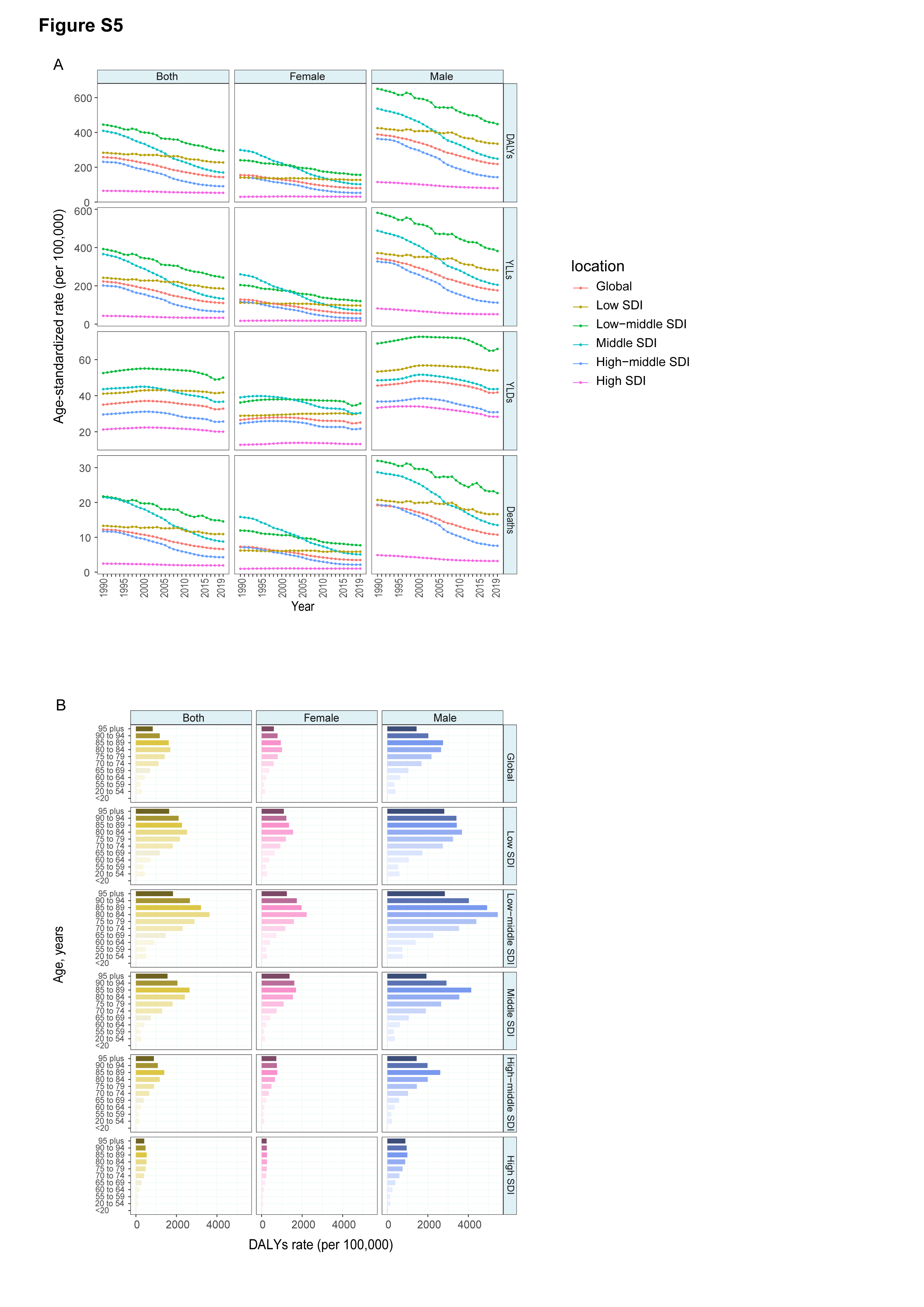

Supplement: Supplementary file 2 — Additional file 2: Figure S1. Age-standardized rate of SEV of 8 main risk factors by SDI quintiles and sex from 1990 to 2019. Figure S2. Contributions of 8 main risk factors to the PAF of age-standardized death due to chronic obstructive pulmonary disease by different SDI quintiles and sexes from 1990 to 2019. Figure S3. Contributions of 8 main risk factors to the PAF of age-standardized YLD due to chronic obstructive pulmonary disease by different SDI quintiles and sexes from 1990 to 2019. Figure S4. Contributions of 8 main risk factors to the PAF of age-standardized YLL due to chronic obstructive pulmonary disease by different SDI quintiles and sexes from 1990 to 2019. Figure S5. The global burden of COPD attributable to occupational particles over the past 30 years. Figure S6. The global burden of COPD attributable to secondhand smoke over the past 30 years. Figure S7. The global burden of COPD attributable to ambient ozone pollution over the past 30 years. Figure S8. The global burden of COPD attributable to high temperature over the past 30 years. Figure S9. The global burden of COPD attributable to low temperature over the past 30 years. [file 12931_2022_2011_MOESM2_ESM.zip › Fig S5/Figure S5_1.tif]

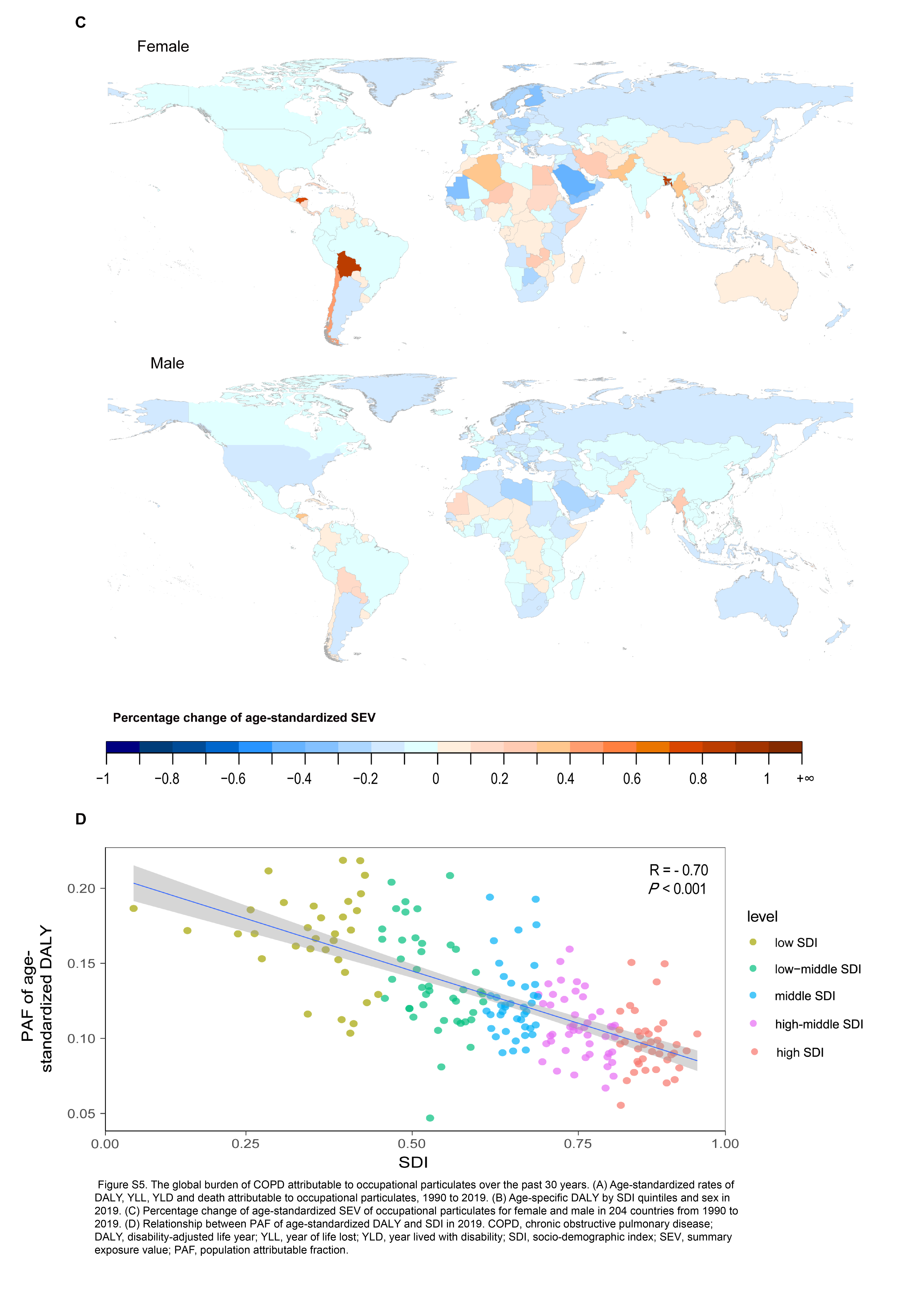

Supplement: Supplementary file 2 — Additional file 2: Figure S1. Age-standardized rate of SEV of 8 main risk factors by SDI quintiles and sex from 1990 to 2019. Figure S2. Contributions of 8 main risk factors to the PAF of age-standardized death due to chronic obstructive pulmonary disease by different SDI quintiles and sexes from 1990 to 2019. Figure S3. Contributions of 8 main risk factors to the PAF of age-standardized YLD due to chronic obstructive pulmonary disease by different SDI quintiles and sexes from 1990 to 2019. Figure S4. Contributions of 8 main risk factors to the PAF of age-standardized YLL due to chronic obstructive pulmonary disease by different SDI quintiles and sexes from 1990 to 2019. Figure S5. The global burden of COPD attributable to occupational particles over the past 30 years. Figure S6. The global burden of COPD attributable to secondhand smoke over the past 30 years. Figure S7. The global burden of COPD attributable to ambient ozone pollution over the past 30 years. Figure S8. The global burden of COPD attributable to high temperature over the past 30 years. Figure S9. The global burden of COPD attributable to low temperature over the past 30 years. [file 12931_2022_2011_MOESM2_ESM.zip › Fig S5/Figure S5_2.tif]

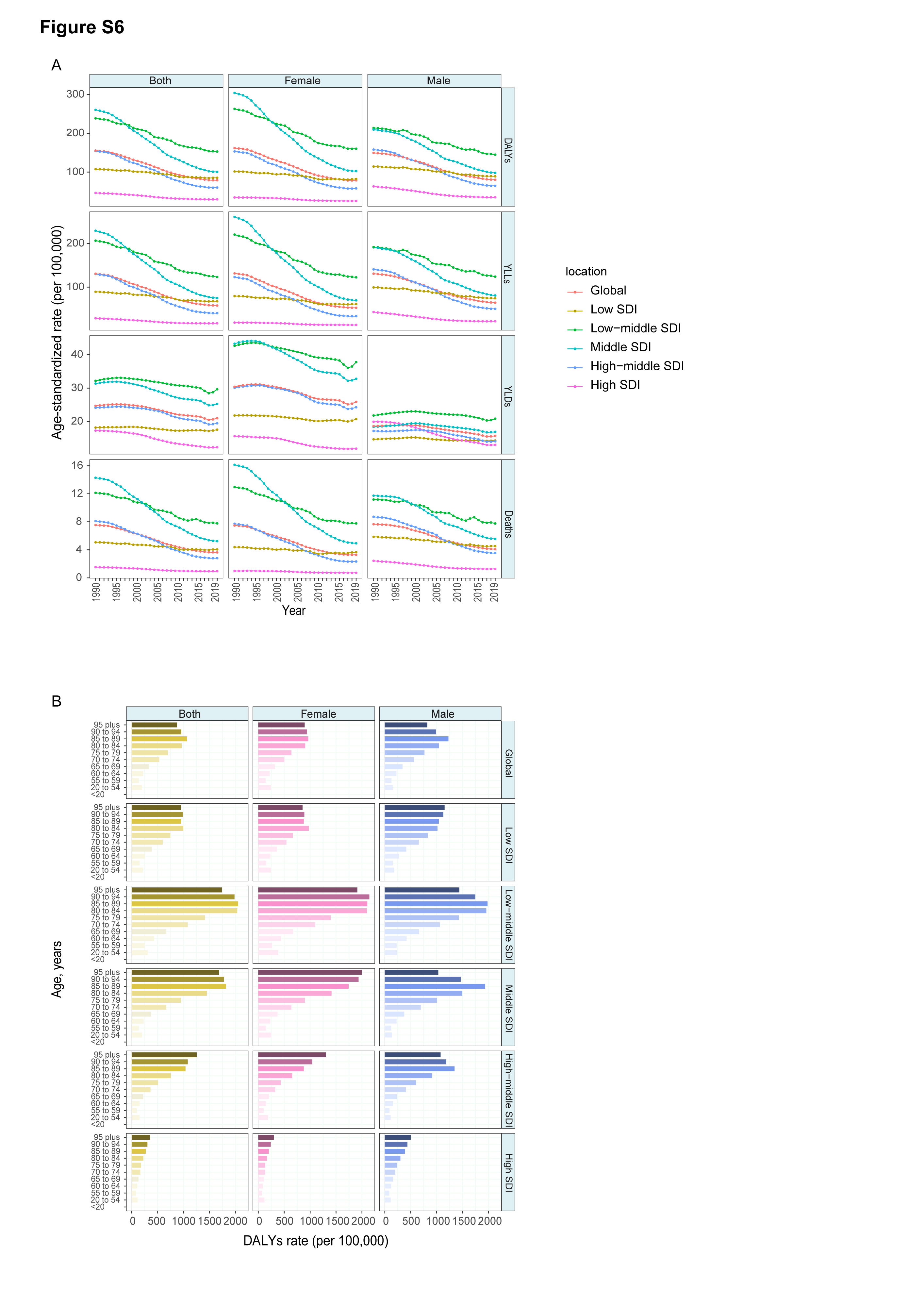

Supplement: Supplementary file 2 — Additional file 2: Figure S1. Age-standardized rate of SEV of 8 main risk factors by SDI quintiles and sex from 1990 to 2019. Figure S2. Contributions of 8 main risk factors to the PAF of age-standardized death due to chronic obstructive pulmonary disease by different SDI quintiles and sexes from 1990 to 2019. Figure S3. Contributions of 8 main risk factors to the PAF of age-standardized YLD due to chronic obstructive pulmonary disease by different SDI quintiles and sexes from 1990 to 2019. Figure S4. Contributions of 8 main risk factors to the PAF of age-standardized YLL due to chronic obstructive pulmonary disease by different SDI quintiles and sexes from 1990 to 2019. Figure S5. The global burden of COPD attributable to occupational particles over the past 30 years. Figure S6. The global burden of COPD attributable to secondhand smoke over the past 30 years. Figure S7. The global burden of COPD attributable to ambient ozone pollution over the past 30 years. Figure S8. The global burden of COPD attributable to high temperature over the past 30 years. Figure S9. The global burden of COPD attributable to low temperature over the past 30 years. [file 12931_2022_2011_MOESM2_ESM.zip › Fig S6/Figure S6_1.tif]

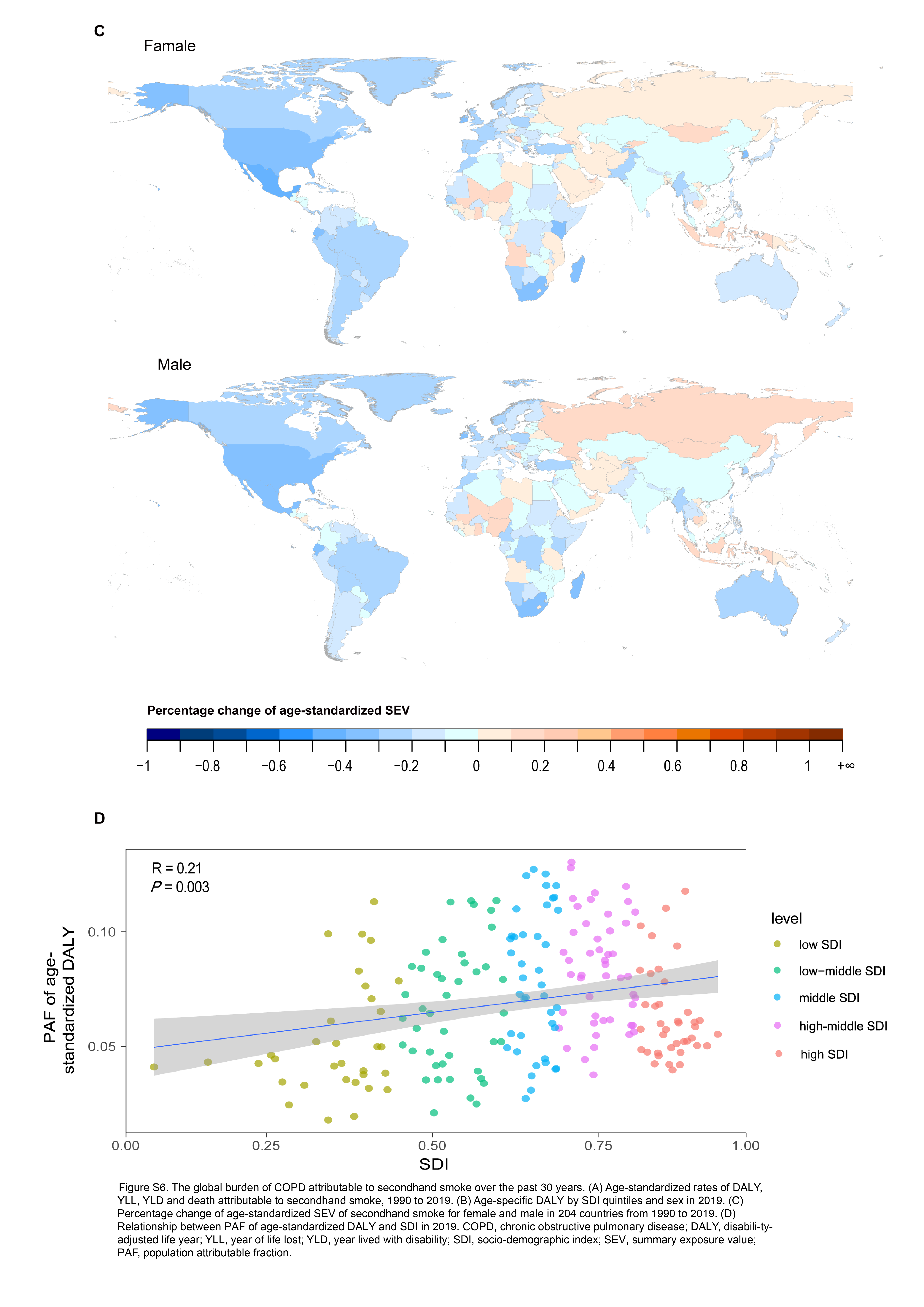

Supplement: Supplementary file 2 — Additional file 2: Figure S1. Age-standardized rate of SEV of 8 main risk factors by SDI quintiles and sex from 1990 to 2019. Figure S2. Contributions of 8 main risk factors to the PAF of age-standardized death due to chronic obstructive pulmonary disease by different SDI quintiles and sexes from 1990 to 2019. Figure S3. Contributions of 8 main risk factors to the PAF of age-standardized YLD due to chronic obstructive pulmonary disease by different SDI quintiles and sexes from 1990 to 2019. Figure S4. Contributions of 8 main risk factors to the PAF of age-standardized YLL due to chronic obstructive pulmonary disease by different SDI quintiles and sexes from 1990 to 2019. Figure S5. The global burden of COPD attributable to occupational particles over the past 30 years. Figure S6. The global burden of COPD attributable to secondhand smoke over the past 30 years. Figure S7. The global burden of COPD attributable to ambient ozone pollution over the past 30 years. Figure S8. The global burden of COPD attributable to high temperature over the past 30 years. Figure S9. The global burden of COPD attributable to low temperature over the past 30 years. [file 12931_2022_2011_MOESM2_ESM.zip › Fig S6/Figure S6_2.tif]

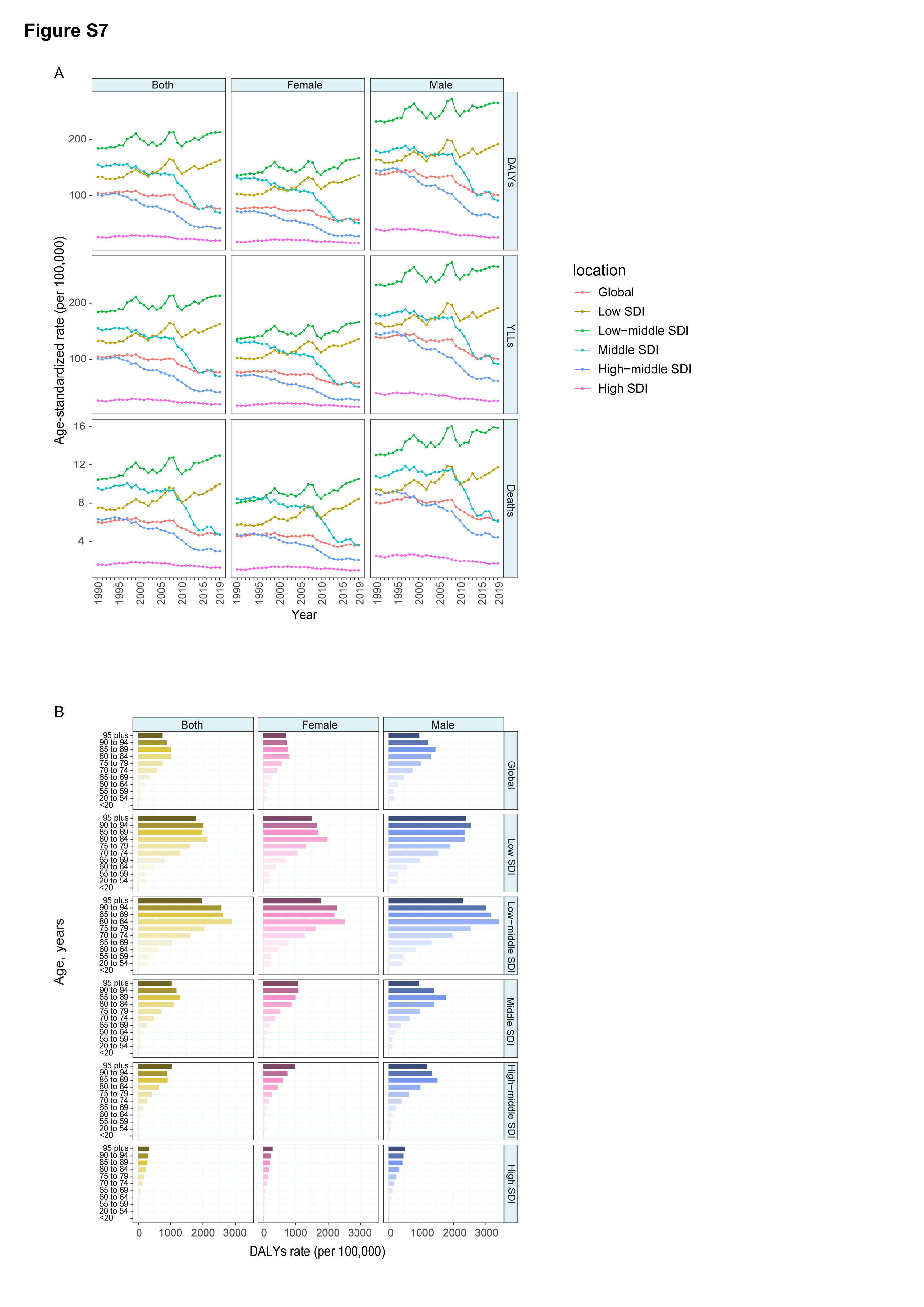

Supplement: Supplementary file 2 — Additional file 2: Figure S1. Age-standardized rate of SEV of 8 main risk factors by SDI quintiles and sex from 1990 to 2019. Figure S2. Contributions of 8 main risk factors to the PAF of age-standardized death due to chronic obstructive pulmonary disease by different SDI quintiles and sexes from 1990 to 2019. Figure S3. Contributions of 8 main risk factors to the PAF of age-standardized YLD due to chronic obstructive pulmonary disease by different SDI quintiles and sexes from 1990 to 2019. Figure S4. Contributions of 8 main risk factors to the PAF of age-standardized YLL due to chronic obstructive pulmonary disease by different SDI quintiles and sexes from 1990 to 2019. Figure S5. The global burden of COPD attributable to occupational particles over the past 30 years. Figure S6. The global burden of COPD attributable to secondhand smoke over the past 30 years. Figure S7. The global burden of COPD attributable to ambient ozone pollution over the past 30 years. Figure S8. The global burden of COPD attributable to high temperature over the past 30 years. Figure S9. The global burden of COPD attributable to low temperature over the past 30 years. [file 12931_2022_2011_MOESM2_ESM.zip › Fig. S7/Figure S7_1.tif]

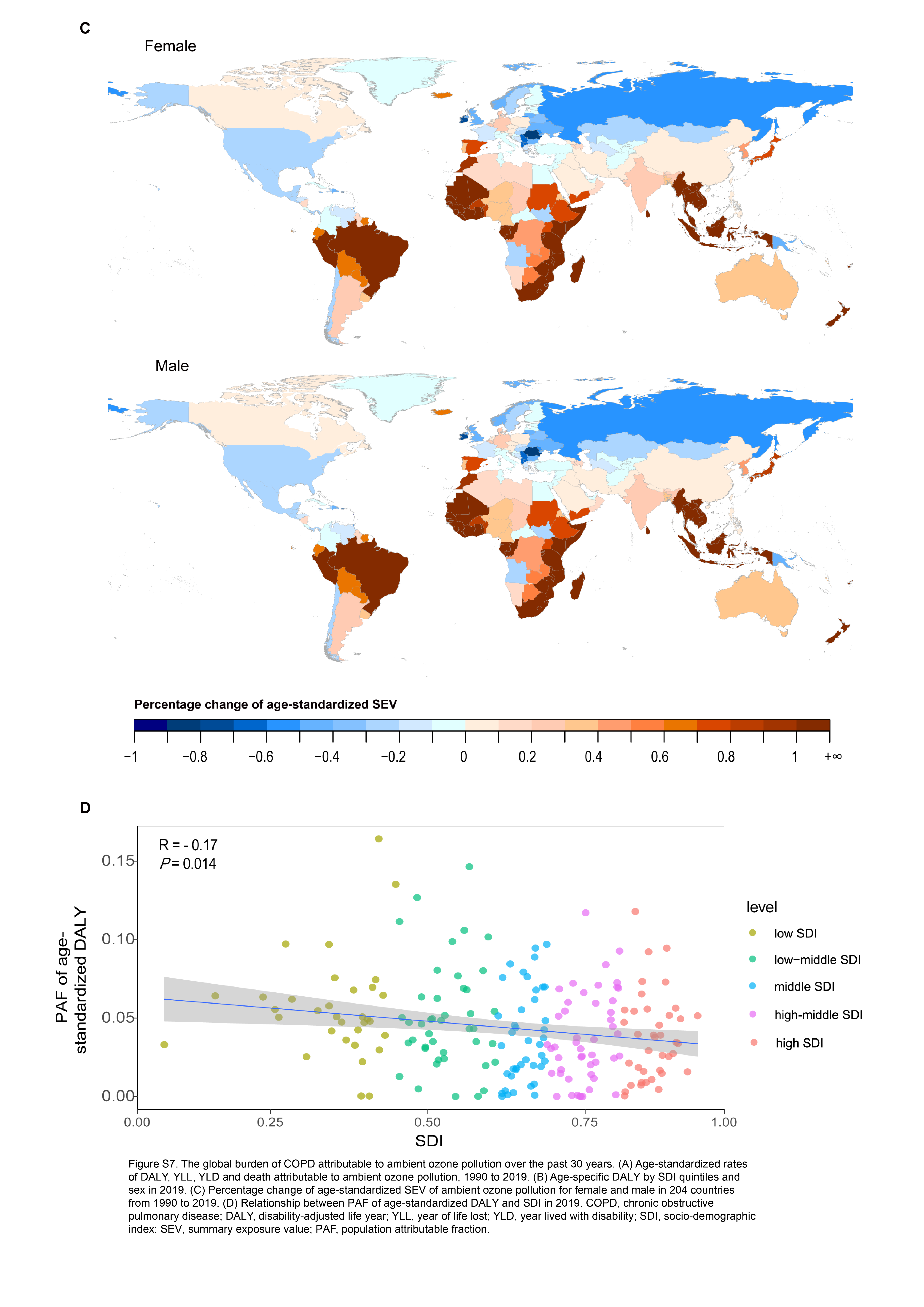

Supplement: Supplementary file 2 — Additional file 2: Figure S1. Age-standardized rate of SEV of 8 main risk factors by SDI quintiles and sex from 1990 to 2019. Figure S2. Contributions of 8 main risk factors to the PAF of age-standardized death due to chronic obstructive pulmonary disease by different SDI quintiles and sexes from 1990 to 2019. Figure S3. Contributions of 8 main risk factors to the PAF of age-standardized YLD due to chronic obstructive pulmonary disease by different SDI quintiles and sexes from 1990 to 2019. Figure S4. Contributions of 8 main risk factors to the PAF of age-standardized YLL due to chronic obstructive pulmonary disease by different SDI quintiles and sexes from 1990 to 2019. Figure S5. The global burden of COPD attributable to occupational particles over the past 30 years. Figure S6. The global burden of COPD attributable to secondhand smoke over the past 30 years. Figure S7. The global burden of COPD attributable to ambient ozone pollution over the past 30 years. Figure S8. The global burden of COPD attributable to high temperature over the past 30 years. Figure S9. The global burden of COPD attributable to low temperature over the past 30 years. [file 12931_2022_2011_MOESM2_ESM.zip › Fig. S7/Figure S7_2.tif]

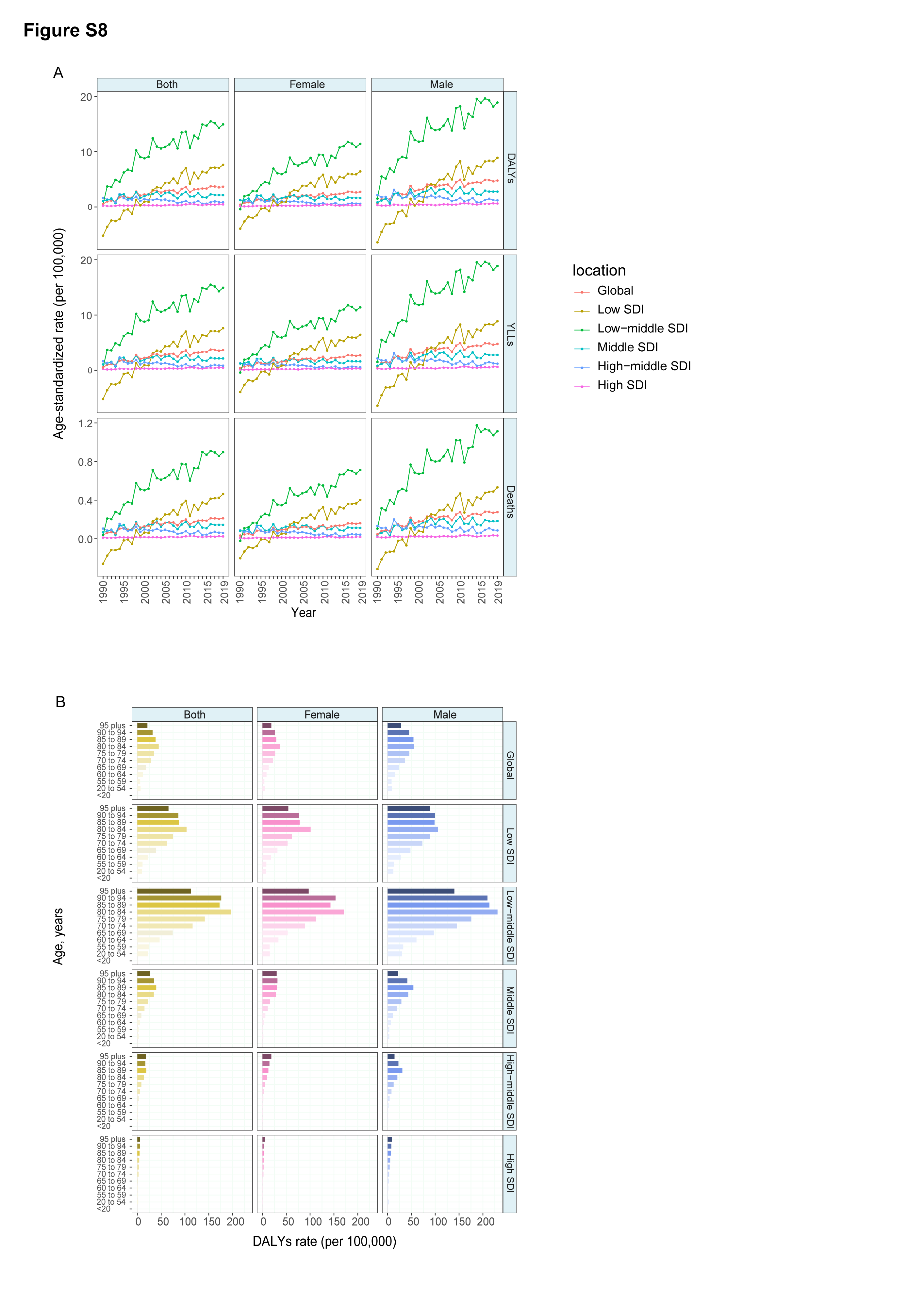

Supplement: Supplementary file 2 — Additional file 2: Figure S1. Age-standardized rate of SEV of 8 main risk factors by SDI quintiles and sex from 1990 to 2019. Figure S2. Contributions of 8 main risk factors to the PAF of age-standardized death due to chronic obstructive pulmonary disease by different SDI quintiles and sexes from 1990 to 2019. Figure S3. Contributions of 8 main risk factors to the PAF of age-standardized YLD due to chronic obstructive pulmonary disease by different SDI quintiles and sexes from 1990 to 2019. Figure S4. Contributions of 8 main risk factors to the PAF of age-standardized YLL due to chronic obstructive pulmonary disease by different SDI quintiles and sexes from 1990 to 2019. Figure S5. The global burden of COPD attributable to occupational particles over the past 30 years. Figure S6. The global burden of COPD attributable to secondhand smoke over the past 30 years. Figure S7. The global burden of COPD attributable to ambient ozone pollution over the past 30 years. Figure S8. The global burden of COPD attributable to high temperature over the past 30 years. Figure S9. The global burden of COPD attributable to low temperature over the past 30 years. [file 12931_2022_2011_MOESM2_ESM.zip › Fig. S8/Figure S8_1.tif]

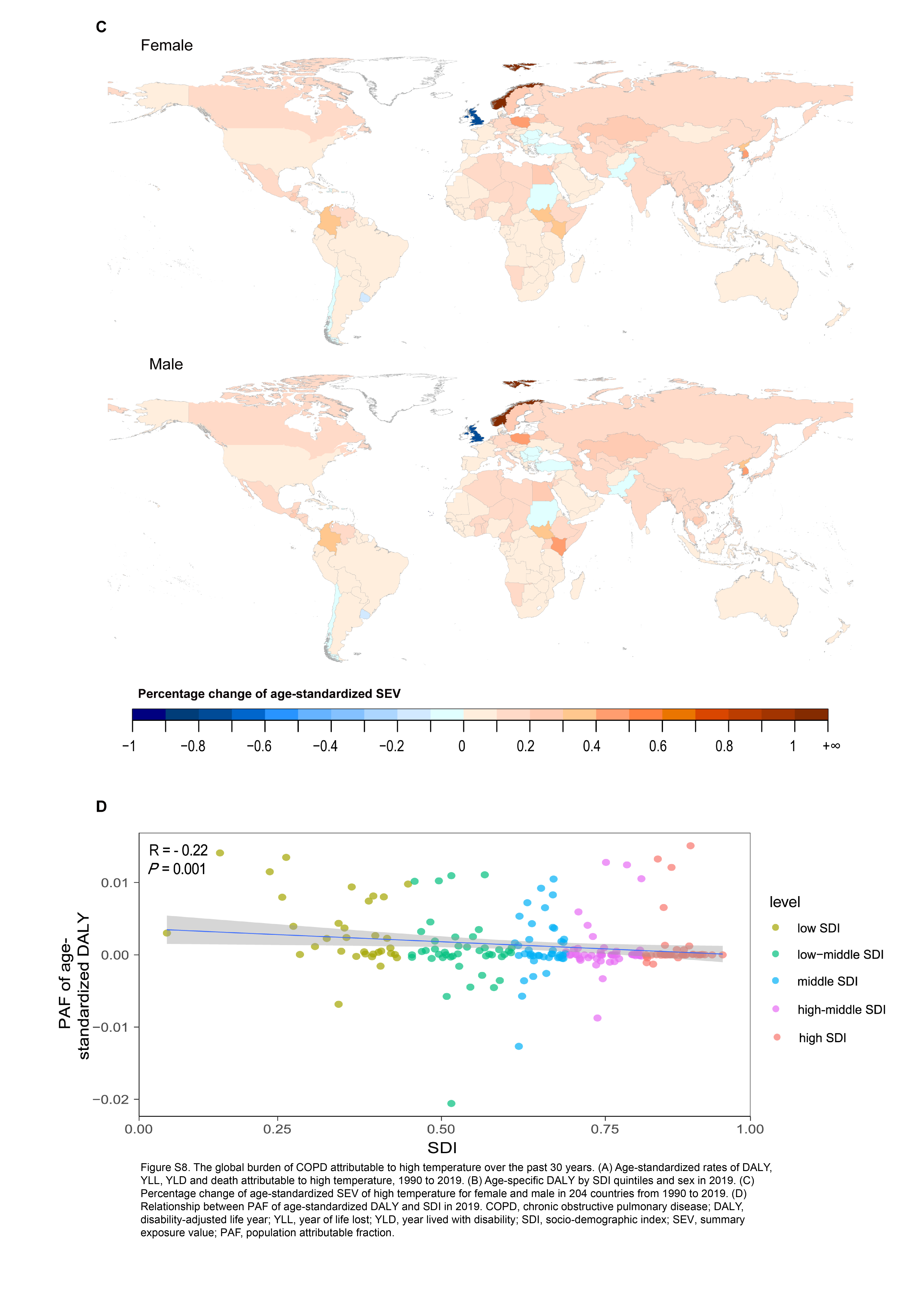

Supplement: Supplementary file 2 — Additional file 2: Figure S1. Age-standardized rate of SEV of 8 main risk factors by SDI quintiles and sex from 1990 to 2019. Figure S2. Contributions of 8 main risk factors to the PAF of age-standardized death due to chronic obstructive pulmonary disease by different SDI quintiles and sexes from 1990 to 2019. Figure S3. Contributions of 8 main risk factors to the PAF of age-standardized YLD due to chronic obstructive pulmonary disease by different SDI quintiles and sexes from 1990 to 2019. Figure S4. Contributions of 8 main risk factors to the PAF of age-standardized YLL due to chronic obstructive pulmonary disease by different SDI quintiles and sexes from 1990 to 2019. Figure S5. The global burden of COPD attributable to occupational particles over the past 30 years. Figure S6. The global burden of COPD attributable to secondhand smoke over the past 30 years. Figure S7. The global burden of COPD attributable to ambient ozone pollution over the past 30 years. Figure S8. The global burden of COPD attributable to high temperature over the past 30 years. Figure S9. The global burden of COPD attributable to low temperature over the past 30 years. [file 12931_2022_2011_MOESM2_ESM.zip › Fig. S8/Figure S8_2.tif]

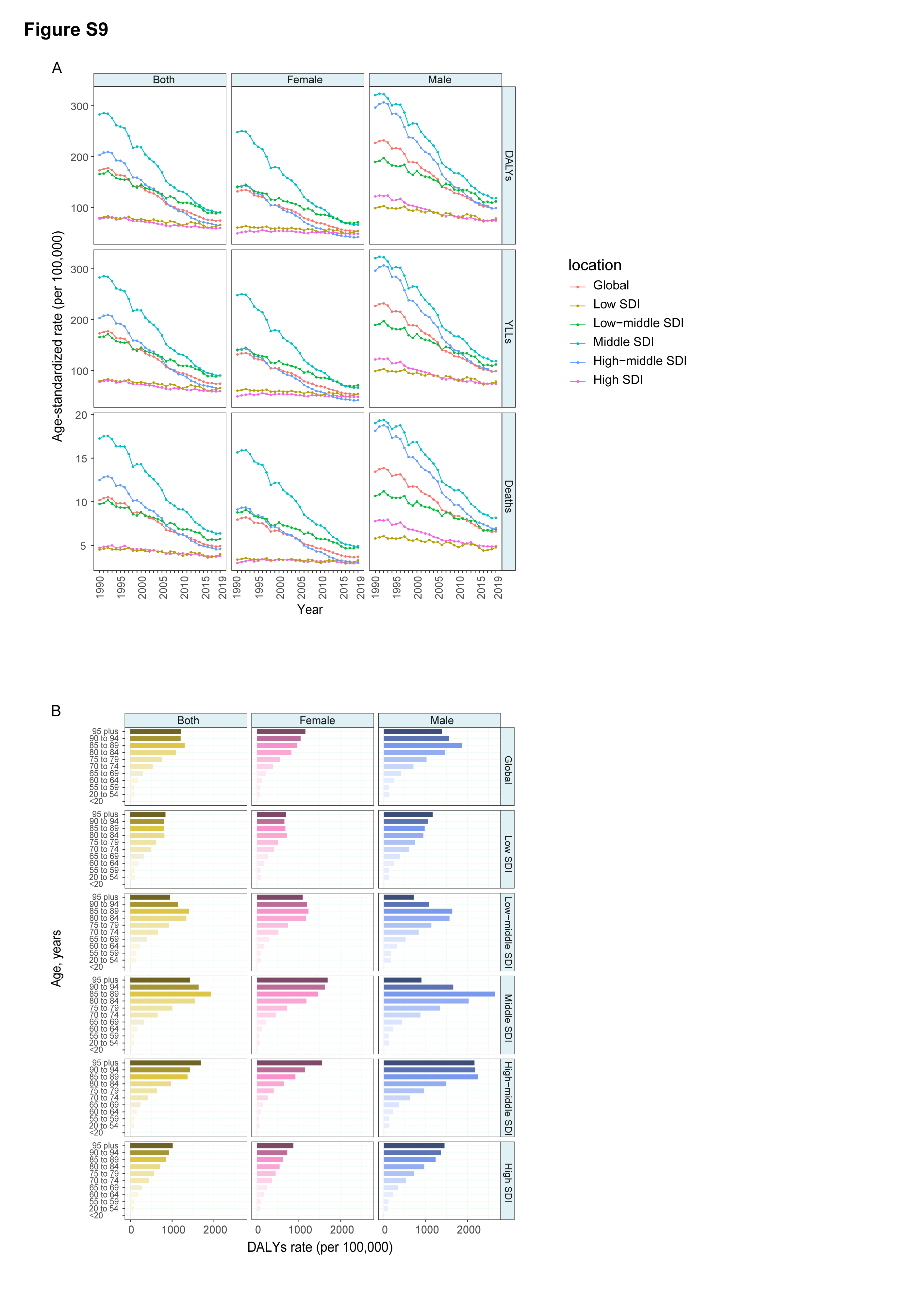

Supplement: Supplementary file 2 — Additional file 2: Figure S1. Age-standardized rate of SEV of 8 main risk factors by SDI quintiles and sex from 1990 to 2019. Figure S2. Contributions of 8 main risk factors to the PAF of age-standardized death due to chronic obstructive pulmonary disease by different SDI quintiles and sexes from 1990 to 2019. Figure S3. Contributions of 8 main risk factors to the PAF of age-standardized YLD due to chronic obstructive pulmonary disease by different SDI quintiles and sexes from 1990 to 2019. Figure S4. Contributions of 8 main risk factors to the PAF of age-standardized YLL due to chronic obstructive pulmonary disease by different SDI quintiles and sexes from 1990 to 2019. Figure S5. The global burden of COPD attributable to occupational particles over the past 30 years. Figure S6. The global burden of COPD attributable to secondhand smoke over the past 30 years. Figure S7. The global burden of COPD attributable to ambient ozone pollution over the past 30 years. Figure S8. The global burden of COPD attributable to high temperature over the past 30 years. Figure S9. The global burden of COPD attributable to low temperature over the past 30 years. [file 12931_2022_2011_MOESM2_ESM.zip › Fig. S9/Figure S9_1.tif]

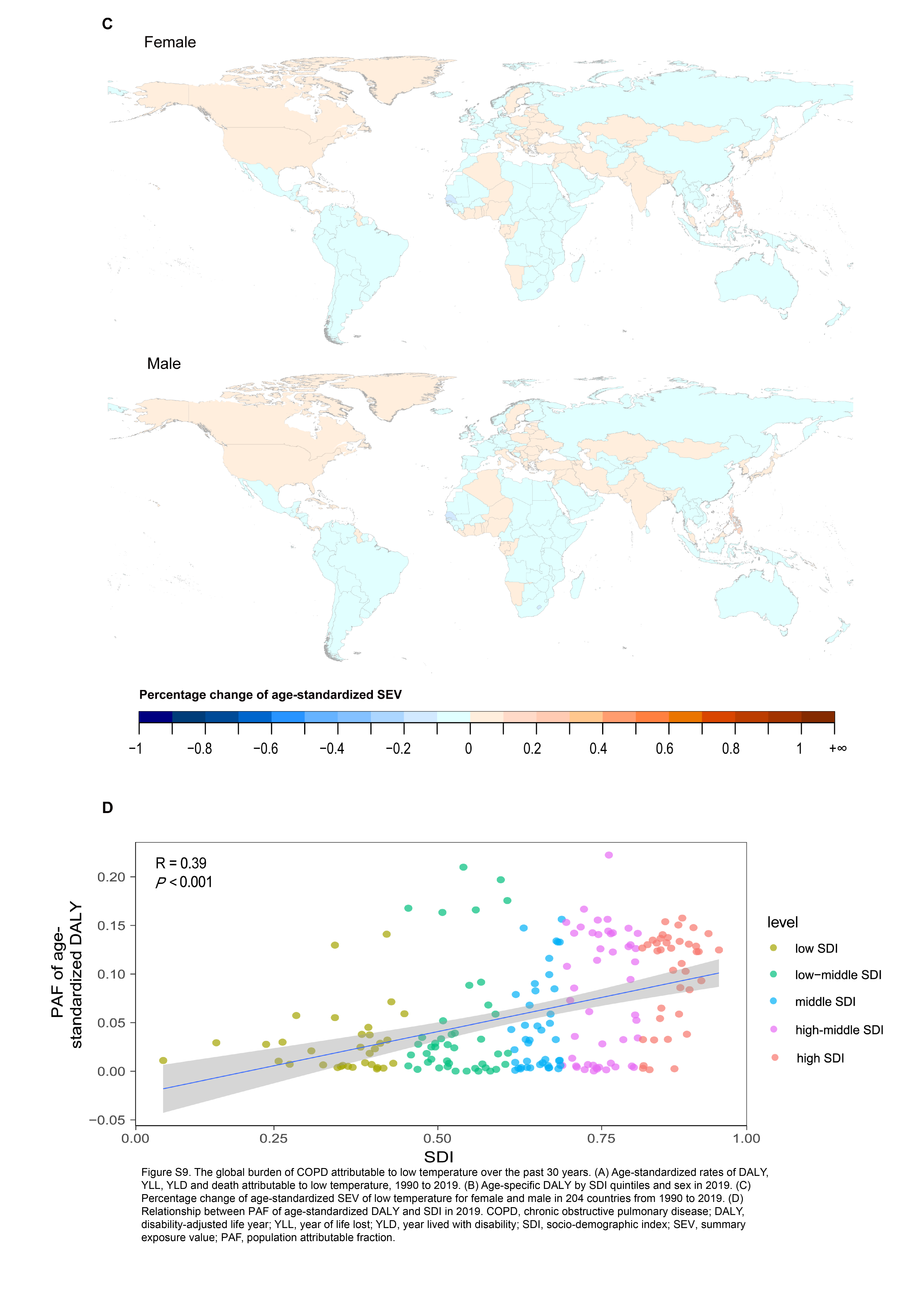

Supplement: Supplementary file 2 — Additional file 2: Figure S1. Age-standardized rate of SEV of 8 main risk factors by SDI quintiles and sex from 1990 to 2019. Figure S2. Contributions of 8 main risk factors to the PAF of age-standardized death due to chronic obstructive pulmonary disease by different SDI quintiles and sexes from 1990 to 2019. Figure S3. Contributions of 8 main risk factors to the PAF of age-standardized YLD due to chronic obstructive pulmonary disease by different SDI quintiles and sexes from 1990 to 2019. Figure S4. Contributions of 8 main risk factors to the PAF of age-standardized YLL due to chronic obstructive pulmonary disease by different SDI quintiles and sexes from 1990 to 2019. Figure S5. The global burden of COPD attributable to occupational particles over the past 30 years. Figure S6. The global burden of COPD attributable to secondhand smoke over the past 30 years. Figure S7. The global burden of COPD attributable to ambient ozone pollution over the past 30 years. Figure S8. The global burden of COPD attributable to high temperature over the past 30 years. Figure S9. The global burden of COPD attributable to low temperature over the past 30 years. [file 12931_2022_2011_MOESM2_ESM.zip › Fig. S9/Figure S9_2.tif]

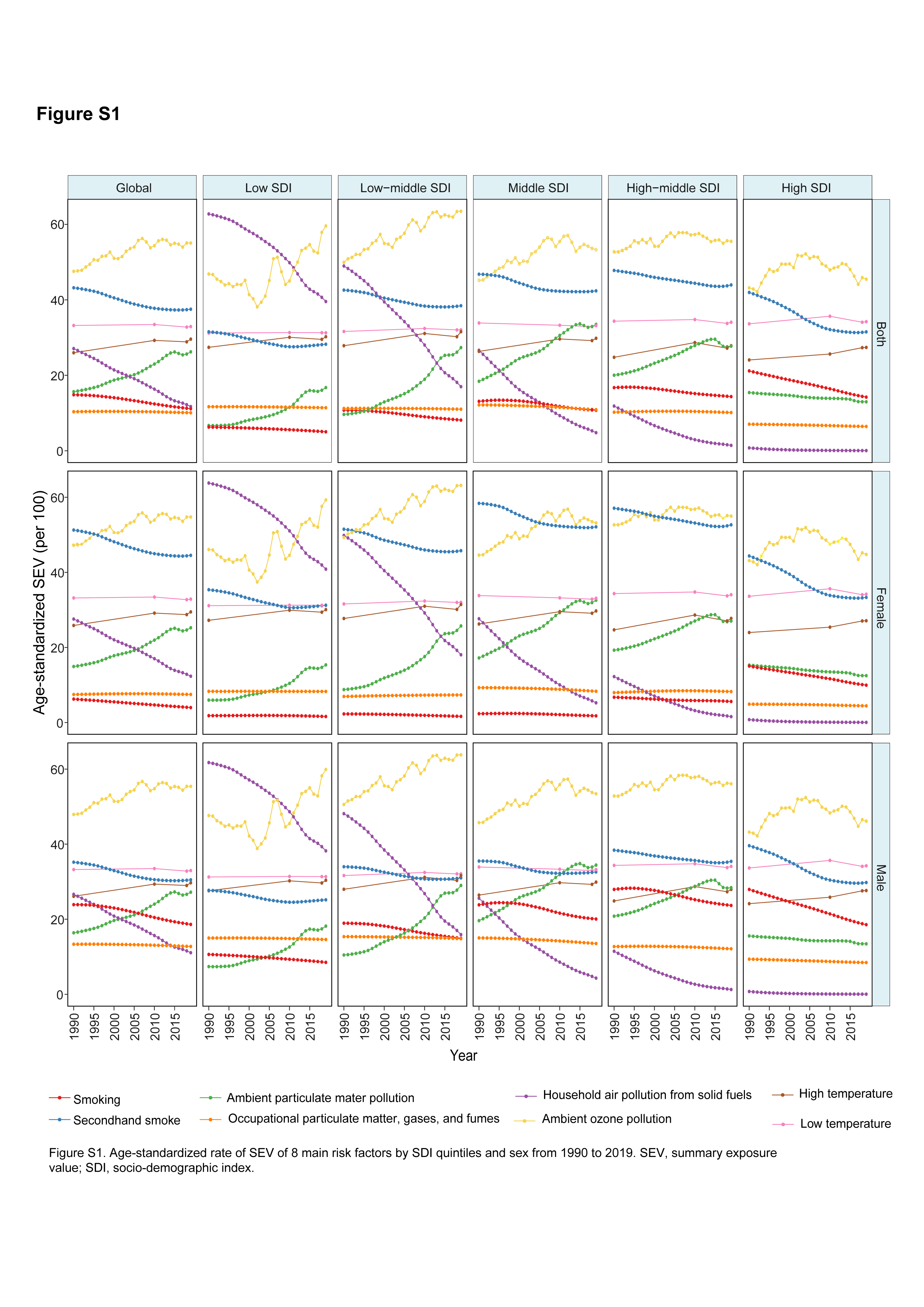

Supplement: Supplementary file 2 — Additional file 2: Figure S1. Age-standardized rate of SEV of 8 main risk factors by SDI quintiles and sex from 1990 to 2019. Figure S2. Contributions of 8 main risk factors to the PAF of age-standardized death due to chronic obstructive pulmonary disease by different SDI quintiles and sexes from 1990 to 2019. Figure S3. Contributions of 8 main risk factors to the PAF of age-standardized YLD due to chronic obstructive pulmonary disease by different SDI quintiles and sexes from 1990 to 2019. Figure S4. Contributions of 8 main risk factors to the PAF of age-standardized YLL due to chronic obstructive pulmonary disease by different SDI quintiles and sexes from 1990 to 2019. Figure S5. The global burden of COPD attributable to occupational particles over the past 30 years. Figure S6. The global burden of COPD attributable to secondhand smoke over the past 30 years. Figure S7. The global burden of COPD attributable to ambient ozone pollution over the past 30 years. Figure S8. The global burden of COPD attributable to high temperature over the past 30 years. Figure S9. The global burden of COPD attributable to low temperature over the past 30 years. [file 12931_2022_2011_MOESM2_ESM.zip › Figure S1.tif]

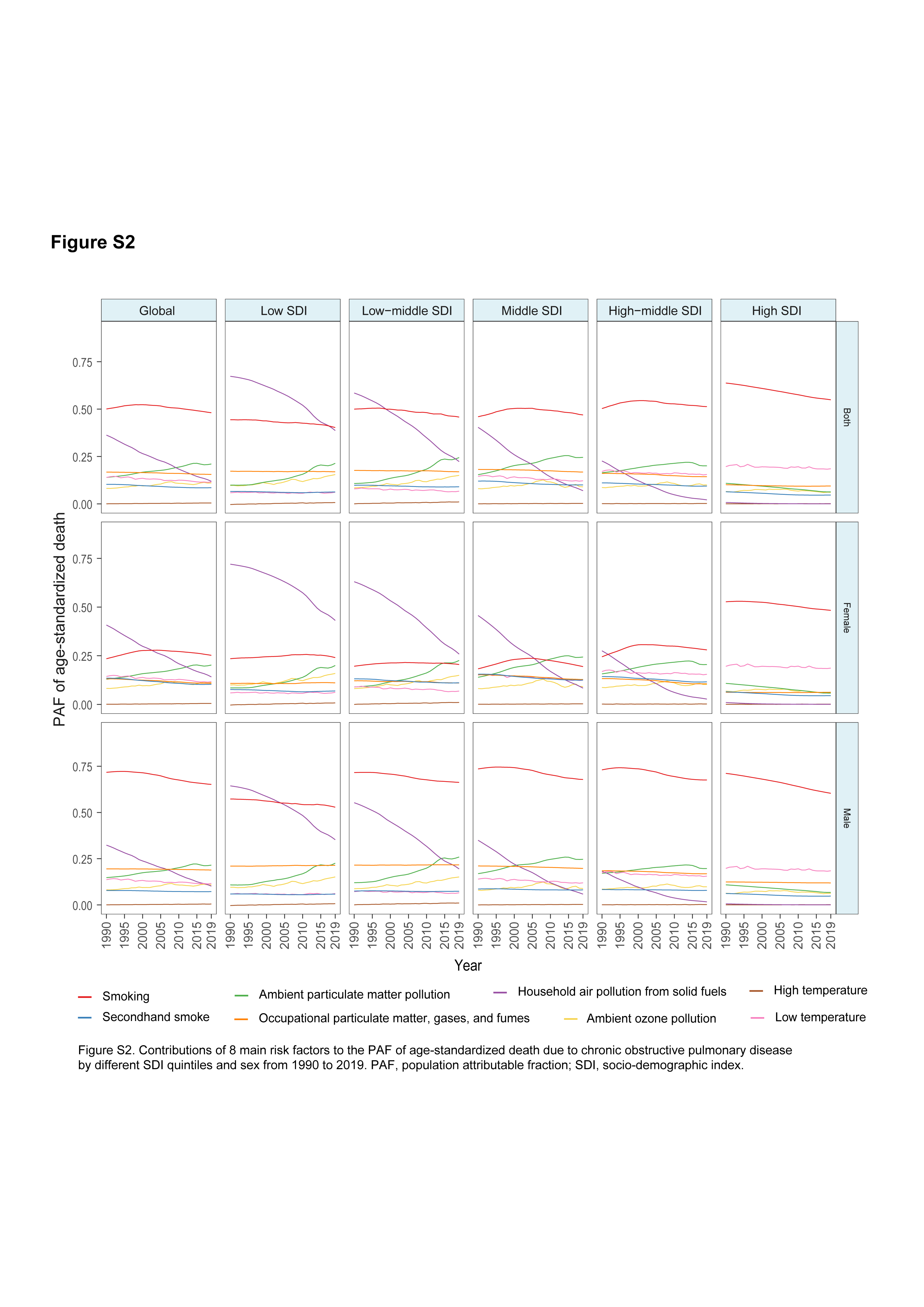

Supplement: Supplementary file 2 — Additional file 2: Figure S1. Age-standardized rate of SEV of 8 main risk factors by SDI quintiles and sex from 1990 to 2019. Figure S2. Contributions of 8 main risk factors to the PAF of age-standardized death due to chronic obstructive pulmonary disease by different SDI quintiles and sexes from 1990 to 2019. Figure S3. Contributions of 8 main risk factors to the PAF of age-standardized YLD due to chronic obstructive pulmonary disease by different SDI quintiles and sexes from 1990 to 2019. Figure S4. Contributions of 8 main risk factors to the PAF of age-standardized YLL due to chronic obstructive pulmonary disease by different SDI quintiles and sexes from 1990 to 2019. Figure S5. The global burden of COPD attributable to occupational particles over the past 30 years. Figure S6. The global burden of COPD attributable to secondhand smoke over the past 30 years. Figure S7. The global burden of COPD attributable to ambient ozone pollution over the past 30 years. Figure S8. The global burden of COPD attributable to high temperature over the past 30 years. Figure S9. The global burden of COPD attributable to low temperature over the past 30 years. [file 12931_2022_2011_MOESM2_ESM.zip › Figure S2.tif]

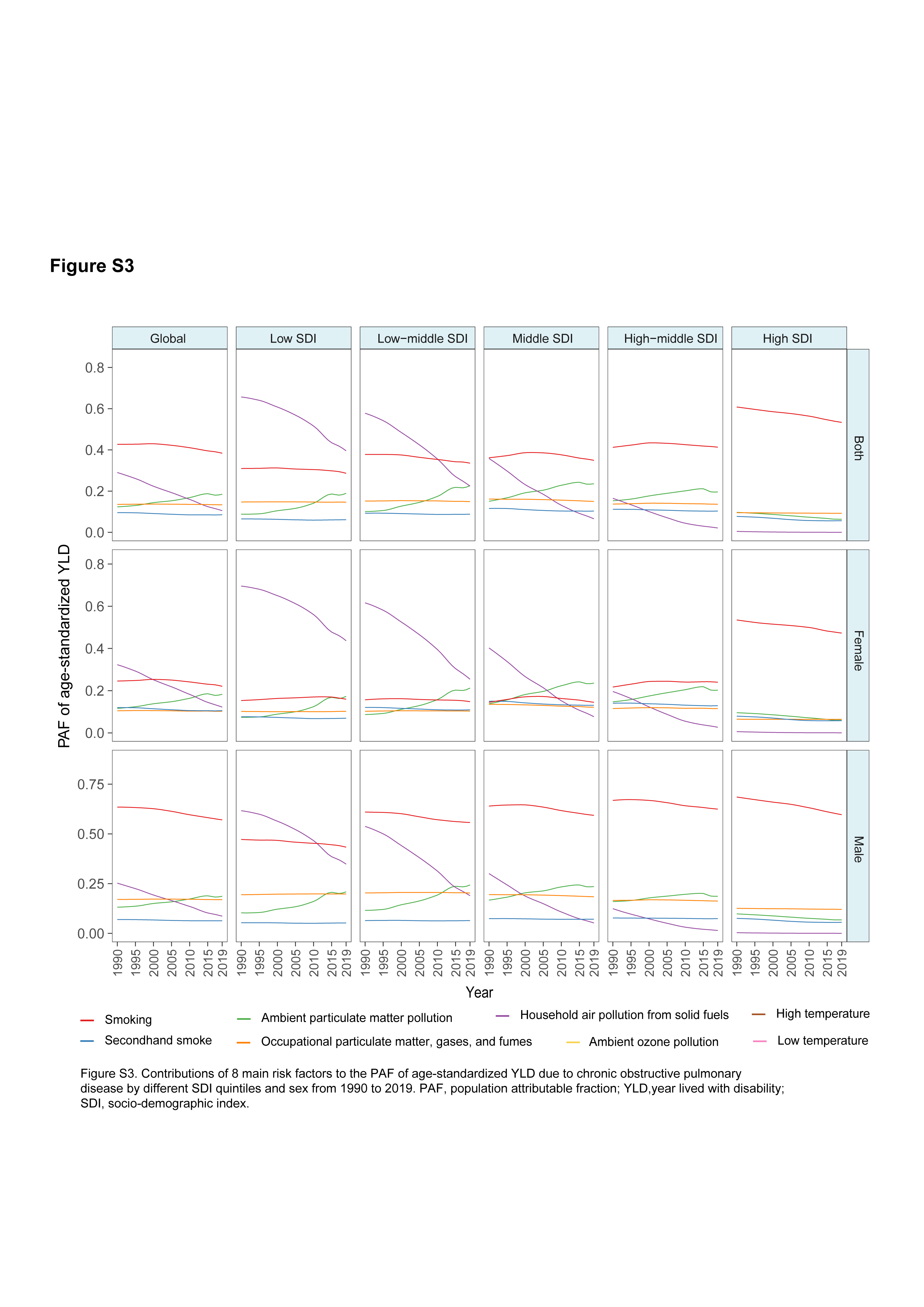

Supplement: Supplementary file 2 — Additional file 2: Figure S1. Age-standardized rate of SEV of 8 main risk factors by SDI quintiles and sex from 1990 to 2019. Figure S2. Contributions of 8 main risk factors to the PAF of age-standardized death due to chronic obstructive pulmonary disease by different SDI quintiles and sexes from 1990 to 2019. Figure S3. Contributions of 8 main risk factors to the PAF of age-standardized YLD due to chronic obstructive pulmonary disease by different SDI quintiles and sexes from 1990 to 2019. Figure S4. Contributions of 8 main risk factors to the PAF of age-standardized YLL due to chronic obstructive pulmonary disease by different SDI quintiles and sexes from 1990 to 2019. Figure S5. The global burden of COPD attributable to occupational particles over the past 30 years. Figure S6. The global burden of COPD attributable to secondhand smoke over the past 30 years. Figure S7. The global burden of COPD attributable to ambient ozone pollution over the past 30 years. Figure S8. The global burden of COPD attributable to high temperature over the past 30 years. Figure S9. The global burden of COPD attributable to low temperature over the past 30 years. [file 12931_2022_2011_MOESM2_ESM.zip › Figure S3.tif]

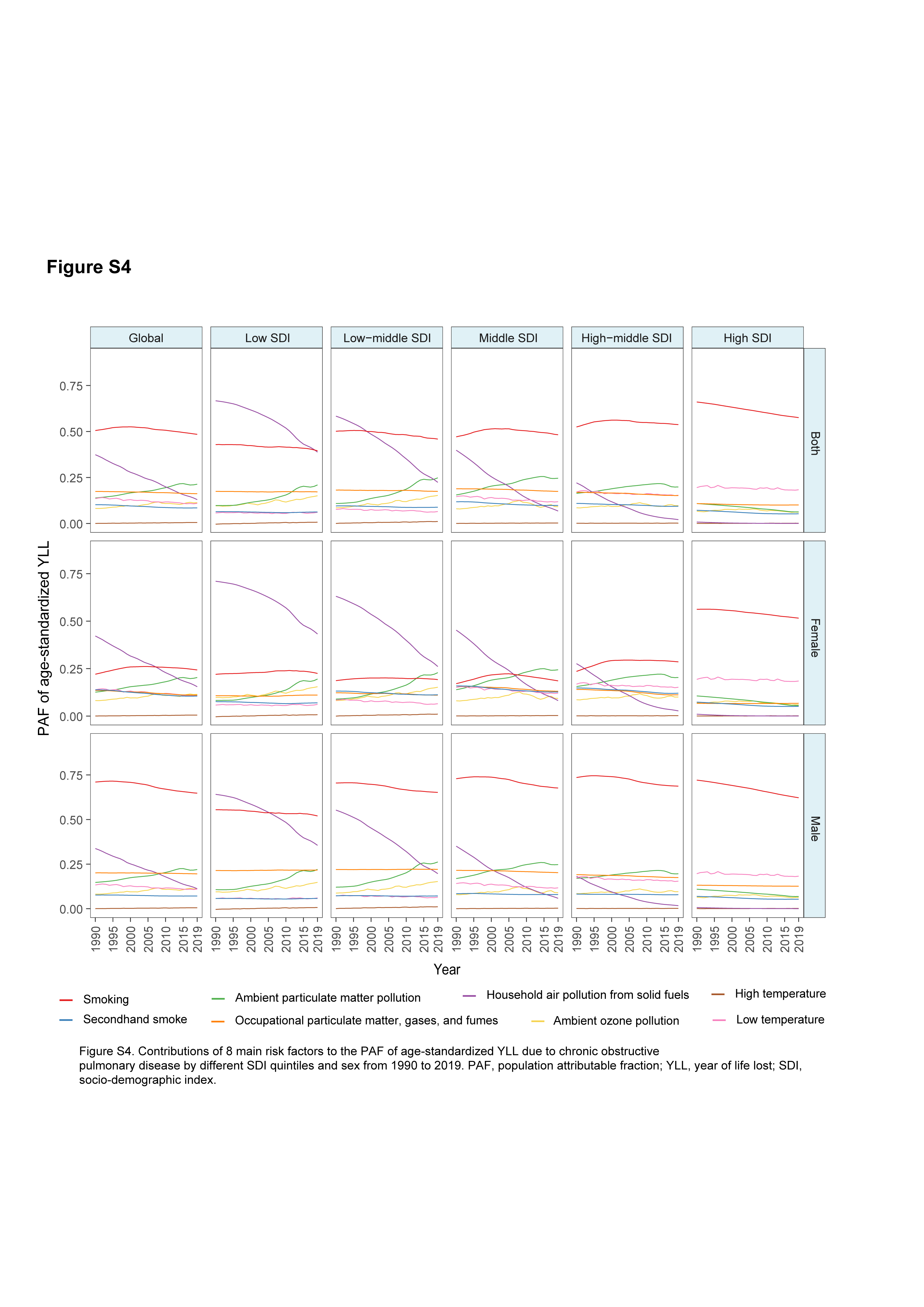

Supplement: Supplementary file 2 — Additional file 2: Figure S1. Age-standardized rate of SEV of 8 main risk factors by SDI quintiles and sex from 1990 to 2019. Figure S2. Contributions of 8 main risk factors to the PAF of age-standardized death due to chronic obstructive pulmonary disease by different SDI quintiles and sexes from 1990 to 2019. Figure S3. Contributions of 8 main risk factors to the PAF of age-standardized YLD due to chronic obstructive pulmonary disease by different SDI quintiles and sexes from 1990 to 2019. Figure S4. Contributions of 8 main risk factors to the PAF of age-standardized YLL due to chronic obstructive pulmonary disease by different SDI quintiles and sexes from 1990 to 2019. Figure S5. The global burden of COPD attributable to occupational particles over the past 30 years. Figure S6. The global burden of COPD attributable to secondhand smoke over the past 30 years. Figure S7. The global burden of COPD attributable to ambient ozone pollution over the past 30 years. Figure S8. The global burden of COPD attributable to high temperature over the past 30 years. Figure S9. The global burden of COPD attributable to low temperature over the past 30 years. [file 12931_2022_2011_MOESM2_ESM.zip › Figure S4.tif]
